# Fungal plant pathogen “mutagenomics” reveals tagged and untagged mutations in *Zymoseptoria tritici* and identifies SSK2 as key morphogenesis and stress-responsive virulence factor

**DOI:** 10.3389/fpls.2023.1140824

**Published:** 2023-05-03

**Authors:** Hannah R. Blyth, Dan Smith, Robert King, Carlos Bayon, Tom Ashfield, Hannah Walpole, Eudri Venter, Rumiana V. Ray, Kostya Kanyuka, Jason J. Rudd

**Affiliations:** ^1^Protecting Crops and the Environment, Rothamsted Research, Harpenden, United Kingdom; ^2^Crop Health and Protection (CHAP), Rothamsted Research, Harpenden, United Kingdom; ^3^Bioimaging Unit, Rothamsted Research, Harpenden, United Kingdom; ^4^Division of Plant and Crop Sciences, School of Biosciences, University of Nottingham, Loughborough, United Kingdom

**Keywords:** Septoria Tritici Blotch, Dothideomycete, *Triticum aestivum*, mitogen activated protein kinase, oxidative stress, genome instability

## Abstract

“Mutagenomics” is the combination of random mutagenesis, phenotypic screening, and whole-genome re-sequencing to uncover all tagged and untagged mutations linked with phenotypic changes in an organism. In this study, we performed a mutagenomics screen on the wheat pathogenic fungus *Zymoseptoria tritici* for altered morphogenetic switching and stress sensitivity phenotypes using *Agrobacterium*-mediated “random” T-DNA mutagenesis (ATMT). Biological screening identified four mutants which were strongly reduced in virulence on wheat. Whole genome re-sequencing defined the positions of the T-DNA insertion events and revealed several unlinked mutations potentially affecting gene functions. Remarkably, two independent reduced virulence mutant strains, with similarly altered stress sensitivities and aberrant hyphal growth phenotypes, were found to have a distinct loss of function mutations in the Zt*SSK2* MAPKKK gene. One mutant strain had a direct T-DNA insertion affecting the predicted protein’s N-terminus, while the other possessed an unlinked frameshift mutation towards the C-terminus. We used genetic complementation to restore both strains’ wild-type (WT) function (virulence, morphogenesis, and stress response). We demonstrated that ZtSSK2 has a non-redundant function with ZtSTE11 in virulence through the biochemical activation of the stress-activated HOG1 MAPK pathway. Moreover, we present data suggesting that SSK2 has a unique role in activating this pathway in response to specific stresses. Finally, dual RNAseq-based transcriptome profiling of WT and SSK2 mutant strains revealed many HOG1-dependent transcriptional changes in the fungus during early infection and suggested that the host response does not discriminate between WT and mutant strains during this early phase. Together these data define new genes implicated in the virulence of the pathogen and emphasise the importance of a whole genome sequencing step in mutagenomic discovery pipelines.

## Introduction

Plant and animal health are consistently threatened by microbial pathogens causing infections and diseases (Fisher et al., 2012). Many (but not all) of these disease-causing agents have relatively small compact genomes and are also haploid in the case of some bacteria and fungi. The next-generation sequencing (NGS) advances now provide highly cost-effective and comprehensive comparative genome analysis for many pathogenic microbes. Combined, these are proving particularly useful in identifying natural genetic variation within populations and allowing genome-wide associated linkage studies to highlight candidate genes related to virulence-associated traits (Zhong et al., 2017; Meile et al., 2018).

Forwards genetics screens performed on pathogenic microbes, including fungal plant pathogens, have used several different methods/approaches. These experiments facilitate an unbiased gene discovery process to dissect the molecular basis of virulence. Nevertheless, before the more recent NGS-based technologies, the genomic analysis of induced experimental mutants was challenging, often reliant upon plasmid rescue or PCR-based approaches to try to identify coding sequences affected by, for example, plasmid insertional screens (Singer & Burke, 2003; Nan & Walbot, 2009). For certain fungal species, the development of *Agrobacterium*-mediated T-DNA-based mutagenesis revolutionised functional gene studies as the procedure generated mostly single insertion events in what are often haploid genomes. Previously the positional analysis of these mutation events required methods such as TAIL-PCR or plasmid rescue to identify candidate genes tagged by T-DNA insertion. Untagged mutations were much more challenging to locate without sequencing technologies. Therefore, the advances in whole genome sequencing have significantly increased the precision of mutation detection in forward genetics experiments resulting in a new field of “mutagenomics” becoming established. This differs from previous approaches in that whole genomes are interrogated in mutant strains/genotypes of plants and microbes to ascertain the full range of all mutations. This approach was first mentioned in the literature by Penna & Jain (2017) and was recently utilised in the model plant *Arabidopsis thaliana* (Hodgens et al., 2020). Mutagenomics enables experimentally generated mutants of pathogens to be genome re-sequenced to ascertain the full spectrum of potentially causative mutations beyond tagged mutations caused by select mutagenesis methods (e.g. T-DNA or plasmid sequence).

The ascomycete fungus *Zymoseptoria tritici* causes the disease Septoria Tritici Blotch (STB) in wheat (*Triticum aestivum*) and is responsible for significant economic losses worldwide. *Z. tritici* has a biphasic infection cycle with an extended symptomless growth phase followed by a switch to a necrotrophic phase when the symptoms of infection begin to show (Goodwin et al., 2011; Steinberg, 2015). The fungus is strictly apoplastic and is not known to invade host cells physically. While interactions between the fungus and wheat leaf cells are not fully understood, hyphal filament extension from “yeast-like” spores is considered essential for successful leaf infection, which occurs predominantly through leaf stomates (Mehrabi et al., 2006b; Motteram et al., 2011; King et al., 2017; Yemelin et al., 2017). This switch between yeast-like and hyphal growth forms is typical of “dimorphic” fungi, and for *Z. tritici in vitro* this depends on nutritional sources and temperature (Rossman et al., 2015). Among the filamentous ascomycetes, this is a unique aspect of *Z. tritici* biology, enabling studies targeting the critical yeast to hyphal growth state switch (Steinberg, 2015; Kilaru et al., 2022). Mutations that affect hyphal growth are lethal to most filamentous fungal pathogens, but in *Z. tritici*, these mutants are culturable in their “yeast-like” form. However, the failure of yeast-like cells to transition into hyphae, or reduction of overall filamentation, results in reduced virulence. A fungus with a lower capacity for stomatal penetration limits the ability for subsequent intercellular colonisation and could severely delay or outright abolish virulence (King et al., 2017; Mohammadi et al., 2020). For this reason, assays that easily identify “switching” mutants are a quick valuable screen for putatively identifying reduced virulence mutants. For *Z. tritici*, the switch to hyphal growth from yeast-like blastospores can be induced and monitored easily following inoculation onto nutrient-poor agar such as tap water agar (TWA) (King et al., 2017).

A reference isolate of *Z. tritici*, IPO323, was one of the first filamentous fungi to have a fully sequenced genome (Testa et al., 2015). The 39.7 Mb genome is arranged in 21 chromosomes, of which the smallest eight (chromosomes 14–21) are mainly considered dispensable for asexual plant infection (Goodwin et al., 2011). With faster, more accurate and cheaper sequencing technologies, further isolates of *Z. tritici* have since been fully sequenced. These include strains 1A5, 1E4, 3D1 and 3D7, all isolated in 1999 from infected wheat fields in Switzerland, as well as single isolates of the related *Zymoseptoria* fungi *Z. brevis*, *Z. pseudotritici* and *Z. ardabiliae* (Lendenmann et al., 2014; Grandaubert et al., 2015; Plissonneau et al., 2018). Work on all these reference strains has illuminated much about the genomics of this important wheat pathogen. *Z. tritici* has been shown to have a high rate of genome plasticity, with regions contributing to low-level “background” mutations occasionally observable within a single strain (Möller et al., 2018; Plissonneau et al., 2018; Oggenfuss et al., 2021). The haploid genome of *Z. tritici* is also amenable to multiple mutagenesis methods, with commonly used and well-established protocols working at different throughputs. *Agrobacterium tumefaciens*-mediated transformation (ATMT) is now widely used for gene deletion, reporter strain generation and studying protein localisation in a targeted manner (Michielse et al., 2005). However, the method can also generate “random” genomic T-DNA insertions to facilitate forward genetics or mutagenomics screens when combined with whole genome re-sequencing.

The global impact of transformation techniques on the genomes of target organisms remains undefined. As previously mentioned, before the advent of and advancements in NGS technologies, researchers used molecular methods to identify the “tagged” sites of genomic integrations (Urban et al., 2015). However, these methods cannot provide information regarding potential “untagged”, spontaneous, or otherwise non-specifically induced mutations that might occur during experimental manipulations and mutagenesis. These untagged mutations can generate a loss of function or missense mutations in proteins and result in observable phenotypic changes. In such cases, a tagged insertion event may unwittingly be ascribed to an impact that an untagged change is causal of. For example, in a transposon tagging experiment in *Arabidopsis thaliana*, the phenotype was attributed to a Ds element jumping more than once in the host genome, impacting multiple sites, and leaving behind a molecular scar (Østergaard & Yanofsky, 2004). Fully re-sequencing genomes from generated mutants enables the detection of any specific “tagged” integration events whilst simultaneously allowing the identification of untagged or “off-target” mutations.

This current study used a whole genome re-sequencing mutagenomics pipeline to investigate T-DNA integrations and untagged mutations in the genomes of five IPO323 mutants, most of which (four) exhibited reduced virulence on wheat. This work identified untagged mutations; some were common amongst the mutant isolates, whereas others were unique to individual isolates. Furthermore, we present and characterise an example of a single gene affected in two of the five mutants, once by direct T-DNA insertion and once by an unlinked Single Nucleotide Polymorphism (SNP) mutation introducing a frameshift. Loss of function of this gene, encoding a putative MAP kinase kinase kinase (MAPKKK) SSK2, imparted many phenotypic changes, including markedly reduced virulence on wheat leaves and differences in responses to stress. Finally, through dual RNAseq, we also present the early *in planta* expressed transcriptome of SSK2 mutants and the comparable host (wheat) transcriptome. These data indicate that whilst SSK2 loss of function reduces the expression of many fungal genes, the host transcriptome does not respond differently to mutants and WT strains, indicating that early responses to the fungus are unchanged. Our study emphasises the key role of whole genome re-sequencing in mutagenomics discovery pipelines and sets a framework for similar studies in other systems.

## Materials and methods

### *In vitro* culture conditions and fungal strains

The reference isolate of *Z. tritici* IPO323, collected in the Netherlands, is the parental strain for the ATMT collection. This strain is virulent towards the experimental wheat cultivar used, cv. Riband.

For all experiments, fungal spores were harvested from 5–6-days-old cultures on Yeast extract peptone dextrose (YPD) agar plates at 16°C. Hygromycin B resistance was conferred by introducing a hygromycin resistance gene cassette as a selectable marker in the original library. To ensure that all mutant isolates were stably transformed, YPD was amended with hygromycin to a final concentration of 100 μg ml^−1^. For later experiments involving complementation mutants, wherein an additional selectable marker was required to enable the selection of positive transformants, a geneticin (G418) resistance gene cassette was introduced. Therefore, hygromycin (100 μg ml^−1^) and geneticin (Sigma Aldrich) to a final working concentration of 200 μg ml^−1^ were added to the YPD media to ensure these isolates were stably transformed.

### *In planta* infection assays and quantitative analysis *via* image analysis

Second leaves of wheat cv. Riband seedlings grown for 21 days in complete compost were inoculated with spores of mutant isolates collected from YPD cultures, which had been washed and re-suspended in dH_2_O+0.01% Tween 20 to a density of 1×10^7^ spores ml^−1^. Infection assays were performed as previously described (Keon et al., 2007). In addition, a wild-type (WT) parental strain of IPO323 was used on each seedling tray to compare the rate of disease symptom development by transformed strains. A minimum of 3 leaves from 3 independent seedlings were tested against each fungal isolate. Additionally, each isolate was tested in this infection assay in at least four replica experiments.

Leaves were photographed periodically, with final photos taken at 21 dpi. For quantitative analysis of disease levels, the LemnaGrid (LemnaTech) software was used as recently described (Chen et al., 2023). In brief, photographs of diseased leaves (TIFs) were imported into the LemnaGrid software. Next, leaves were segmented based on a combination of intensity thresholding and a colour-based classification. Finally, the proportion of diseased tissue for each leaf was quantified using an additional colour-based classification (for chlorotic, necrotic or healthy tissue) which converted data into a percentage of each category for each leaf. Scores were then exported for subsequent quantitative and statistical analysis.

### *In vitro* assays for hyphal growth and stress sensitivity tests

A range of media was used to compare *in vitro* growth phenotypes of *Z. tritici* mutants. For the following assays *in vitro*, blastospores were grown from glycerol stocks and then collected from 5–6-day YPD amended with hygromycin incubated at 16°C. Approximately 1×10^7^ spores ml^−1^ (or a dilution series thereof) were suspended in dH_2_0+0.01% Tween20 and then spotted as drops of 5 μl, allowed to air dry on the agar surface before plates were then sealed with parafilm.

Spore suspensions were also spot-inoculated (as above) onto the surfaces of 1% tap-water agar (TWA) plates for filamentous growth assays. TWA plates were sealed and kept at room temperature. Growth was monitored 2-, 5- and 8-days post-inoculation (dpi). Final macroscopic photographs of hyphal development (radial growth of hyphae from the central spot) were taken at 10 dpi.

YPD media was also amended with various stressors at concentrations used in previous literature (Mehrabi et al., 2006b; Motteram et al., 2011; Yemelin et al., 2017; Francisco et al., 2019). A 200 μg ml^−1^ of calcofluor white was used to induce cell wall integrity stress. To cause oxidative stress, YPD media containing hydrogen peroxide at a concentration of 5 mM. A concentration of 1 M sorbitol was used for osmotic stress conditions. Finally, to test for the resistance of *ZtSsk2* disrupted mutants to the phenylpyrrole fungicide fludioxonil (Fluka), a working concentration of 30 μg ml^−1^ was used. Both YPD and YPD amended plates were sealed and kept at 16°C for 5 days. Growth was monitored from 2 dpi, and final photographs of yeast-like growth levels were taken 5 days post-inoculation (dpi).

### *In planta* inoculation for scanning electron microscopy (SEM)

A different approach was used from the standard attached wheat leaf bioassay to prevent damage to the wheat leaf surface. Instead, a 0.1% Tween20-water solution with a spore concentration of 1×10^7^ spores ml^−1^ was paint brush inoculated onto leaf surfaces.

For the scanning electron microscopy (SEM), approximately 5 mm square regions were cut from the leaf samples and attached to aluminium stubs with a 50:50 mixture of graphite:TissueTek. The samples were frozen in liquid nitrogen and transferred to the GATAN ALTO 2100 cryo prep system. Samples were etched and coated in a thin layer of gold. Micrographs were collected using a JEOL 6360 scanning electron microscope at 5kV.

### Genome re-sequencing and identification of T-DNA insertion sites

Genomic DNA from YPD-grown liquid cultures collected by vacuum filtration was prepared using the Illustra Phytopure DNA extraction kit (GE Healthcare) following the manufacturer’s instructions for all samples and sent for genome re-sequencing by Illumina HiSeq2500 with a 250 bp paired-end read metric and a target of 100× coverage (BGI genomics).

Read quality was assessed using Fastqc (v0.11.9, https://www.bioinformatics.babraham.ac.uk/projects/fastqc/) and Trimmomatic (v0.38-Java-1.8, http://www.usadellab.org/cms/?page=trimmomatic) was used to remove any remaining Illumina adaptors. The position of the T-DNA insertion was determined by mapping paired-end reads to the T-DNA plasmid reference. Where one mate was mapped and the other mate was unmapped, this unmapped read was likely to inform the boundary position of the T-DNA insertion site. A stack of forward reads represents the 5’ T-DNA insertion boundary loci boundary, and the stack of reverse reads represents the 3’ T-DNA insertion boundary loci. The gap between these stacks, therefore, represented any deleted sequence. Copies of T-DNA insertion are determined by the average coverage of mapped reads to the T-DNA versus mapping the raw data to the fungal genome. Detailed instructions on running the FindInsertSeq workflow are available in the previous publication (Urban et al., 2015; https://github.com/Rothamsted/script-collection/tree/master/FindInsertSeq).

### Determination of non-T-DNA mutation sites in *Z. tritici* genomes

Variants (SNPs/indels) were called against the reference IPO323 genome assembly using FreeBayes (v. 1.2.0.4-intel.2019.01). Raw variant counts were filtered using SNPsift (v.1.7.0_161) by the depth equal to, or greater than ten, and a quality score equal to, or greater than, 30. To categorise and assess the likely impacts on annotated genes SNPeff 4.3t (build 2017-11-24 10:18) was used. For high/medium impact variants of interest or concern, PROVEAN (Protein Variation Effect Analyser; http://provean.jcvi.org/index.php) with default settings was also used to predict whether an amino acid substitution or indel impacted the biological function of a protein.

### Agrobacterium-mediated transformation of *Z. tritici*

The original library generation was detailed in Motteram et al. (2011). The vector pCHYG (providing resistance to Hygromycin B) was transformed into an IPO323 isolate by random *Agrobacterium*–mediated transformation using *Agrobacterium* strain AGL-1. For the generation of the complementation construct used in this study the Gibson assembly method and a pCGEN vector, conferring geneticin resistance was used. Primers used in construct building for Gibson assembly were designed using the NEBuilder tool (https://nebuilder.neb.com/). The plasmid was assembled following the Gibson Assembly^®^ Protocol (E5510) and reagents produced by NEB. The generated transformation plasmid was verified by restriction digest using colony PCR of transformed *E. coli* ahead of miniprep. The manufacturer’s protocol for transforming chemically competent cells NEB^®^ 5-alpha Competent *E. coli* (High Efficiency, DH5α) was followed. After overnight incubation, the vector was isolated using a QIAprep spin miniprep kit (Qiagen) following the manufacturer’s instructions.

Electrocompetent AGL-1 *Agrobacterium* cells were transformed following the protocol described in Motteram et al. (2009), using 1 μg of generated pCGGEN+Zt*SSK2* plasmid DNA and the default Agro setting on a BioRad Micropulser. LB Miller agar amended with Kanamycin was used to select for successfully transformed *A. tumefaciens.* Stocks were then made in 50% glycerol and stored at −80°C. *Agrobacterium*-mediated transformation of *Z. tritici* was carried out following a protocol first outlined by Zwiers & De Waard (2001) and further developed by Mehrabi et al. (2006b). Briefly, *Z. tritici* spores and *A. tumefaciens* cells were co-cultivated on cellophane disks on the surface of induction media agar containing acetosyringone. The discs were subsequently transferred to *Aspergillus nidulans* minimal medium agar, which was amended with hygromycin, geneticin and timentin to select stable fungal transformants. Putative transformants were typically observed after 14 days and were subcultured onto YPD agar plates amended with hygromycin or geneticin and timentin. Finally, 50% glycerol stocks were made and stored at −80°C.

### Anti-active MAPK western blotting for oxidative stress responses

*Z. tritici* isolates were grown for 6 days in YPD broth, starting with a single 10 μL loop of blastospores taken from YPD agar. After 6 days of growth shaking in fresh YPD broth, the fungal cultures were “spiked” with 25 mM H_2_O_2_. Fungal cells were collected by vacuum filtration 30 and 60 minutes after exposure and snap-frozen, then stored at −80°C before protein extraction.

Frozen cells were transferred to 2 mL tubes with two small ball bearings and freeze-dried using LyoDry Compact Benchtop Freeze Dryer (MechaTech Systems) overnight for total protein extraction. First, to lyse the samples, Y-PER™ Yeast Protein Extraction Reagent (ThermoScientific) was added. Then, typically for 150 mg fungal material, 1.5 ml was added with a Protease Inhibitor Cocktail (100X) (ThermoScientific). Next, a TissueLyserII was used to agitate and homogenise the mixture for 5 min at 20 Hz. Next, the samples were spun in a centrifuge (14,000 g, 10 min), and the supernatant was transferred to a fresh tube. For storage, 200 μL protein extracts were added to 50 μL 5X SDS loading buffer and kept at −20°C.

Sodium dodecyl sulphate-polyacrylamide gel electrophoresis (SDS-PAGE) protocol was performed as described in Rudd et al. (2008) using 10% resolving gels. Benchmark ladder and MagicMark XP ladder (ThermoFisher) were used for protein size estimations. Following electrophoresis, proteins were blotted onto nitrocellulose membranes (GE Healthcare, 0.1 μm pore size). Correct transfer to the membrane was assessed by staining with ponceau S. The membrane was first washed three times (5 min, RT) in TBS-T buffer before blocking in 5% non-fat milk (Marvel Skimmed Milk Powder) in TBS-Tween buffer. The membrane was rewashed three times (5 min, RT) in TBS-Tween, before subsequent probing and blotting according to the supplier’s guidelines for the Anti-p44/42 and p38 antibodies (Cell Signalling Technologies) using 5% BSA TBS-Tween buffer with the primary antibodies. The secondary antibody, horseradish peroxidase-conjugated goat anti-rabbit antibody (Invitrogen), was used at a 1:10,000 dilution in TBS-T + 5% non-fat milk powder. Finally, chemiluminescence was performed with ECL reagents (GE Healthcare) and blot imaging using an Odyssey Fc imager (Li-Cor), with a 10 min exposure time. Images were optimised and saved using Image Studio 5.2. Protein loading controls were generated by counter-straining membranes with Naphthol blue black (Amido Black).

### RNA sequencing and GO term analyses

The WT IPO323 and T-DNA insertion mutant strain 4-124 were inoculated onto susceptible wheat leaves (cv Riband as previously described). Infected leaf tissues were excised at 6 and 24 h and snap-frozen. Each technical and biological replicate incorporated five leaves. Total RNA was isolated using the TRIZOL procedure (Invitrogen), and RNA quality was assessed using Nanodrop and gel electrophoresis. Directional mRNA polyA enriched library preparation and sequencing were performed by Novogene (Cambridge, UK) using Illumina NovaSeq paired-end 150 bp sequencing.

Quality control of reads was performed using MultiQC. Sequence trimming of recognised adaptors was performed using Trimmomatic (Bolger et al., 2014). Reads were mapped to the *Z. tritici* IPO323 genome using HiSat2 (Kim et al., 2019). Principal Component Analysis (PCA) was performed on sample differences of variance stabilising transformed (vsd) gene count data to confirm that biological replicates clustered together. Count determination was performed using featureCounts (Liao et al., 2014). Library normalisation and differential expression (DE) calling were done using the Bioconductor package DESeq2 (Love et al., 2014; https://bioconductor.org/packages/release/bioc/html/DESeq2.html) in R studio. Gene expression levels were individually compared between WT IPO323 and the 4-124 ATMT mutant samples for each time point. DEGs were identified by applying a log2 fold change filter of ≥ 1 or ≤ −1, and the DESeq2 implementation of Benjamini-Hochberg (Benjamini & Hochberg, 1995) was used to control for multiple testing (FDR<0.05).

Gene Ontology (GO) enrichment analysis was performed for all significantly up-and down-regulated *Z. tritici* genes using OmicsBox to identify the overrepresented GO term BLAST2GO Enrichment. This list of fungal differentially expressed genes was then subjected to PAM (partition around medoids) clustering, with the number of clusters determined using the pamk function in R (*fpc* package version 2.2-9).

The same process was applied to the reads mapping to the Chinese Spring (v1.1) wheat genome, with the exception of a BLAST2GO GO enrichment analysis. To rapidly functionally profile the identified differentially expressed genes g:Prolfiler (https://biit.cs.ut.ee/gprofiler/gost) “g:OSt functional profiling” was performed using default values and ENSEMBL Plants *Triticum aestivum* (taestivum version: IWGSC) (Raudvere et al., 2019).

### KnetMiner knowledge graphs and keyword searches

Zymoseptoria KnetMiner (https://knetminer.com/Zymoseptoria_tritici/) can generate knowledge graphs using *Z. tritici* JGI genome identifiers (e.g. Mycgr3G67344). These graphs identify related proteins, genes, domains, associated phenotypic information and more from databases and broader literature (Hassani-Pak et al., 2021). A prototype Zymoseptoria KnetMiner currently draws on data and literature from a few fungi, including Saccharomyces yeast, *Fusarium graminearum*, *Neurospora crassa* and *Aspergillus nidulans*.

The KnetMiner keyword search function was used to identify a list of candidate genes associated in the literature with “HOG1”, “oxidative stress”, “osmotic stress”, “cell wall integrity”, “hyphal growth”, “filamentous growth”, and “dimorphism” from data from select model fungi and the yeast *S. cerevisiae*. These keyword searches generated a list of 1964 genes “linked” to those search terms, with duplicate entries removed. We compared this to our list of differentially expressed genes from our *in planta* RNA sequencing experiment.

## Results

### *In planta* characterisation of *Agrobacterium tumefaciens* mediated (ATMT) *Z. tritici* transformants

We had previously carried an initial high throughput virulence screen on the library of 631 random ATMT mutants of *Z. tritici* IPO323 as the parental strain for reduced *in planta* infection on the susceptible wheat cv. Riband (Rudd, unpublished). In this initial screen, performed only on single leaves, fourteen transformants were identified as either non-pathogenic or with reduced virulence. The mutants in this library all have Hygromycin B resistance conferred by use of the vector pCHYG for ATMT. Two from this collection had previously been further analysed, describing the impact of mutations detected in the *ZtALG2* (previously published as *MgAlg2*) and *ZtGT2* genes on *Z. tritici* virulence (Motteram et al., 2011; King et al., 2017). From the remaining non-pathogenic or reduced virulence mutants, which had not been further characterised, five isolates are explored herein.

**Figure 1:**
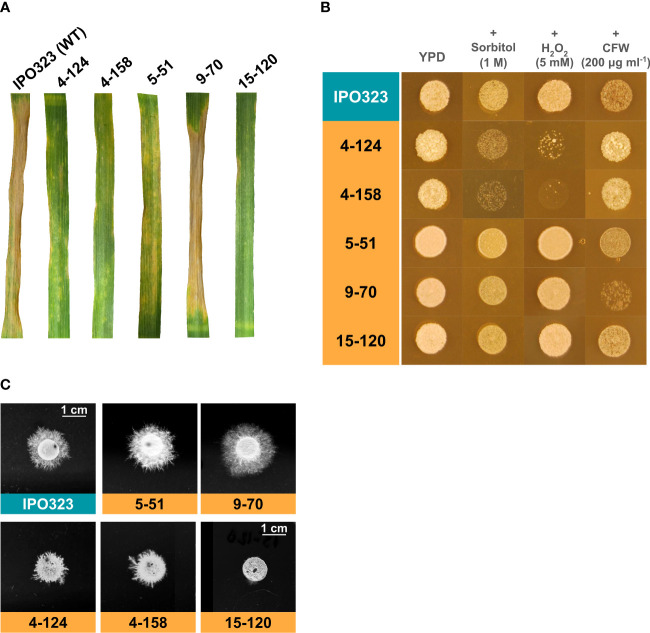
Characterisation of *Z. tritici* T-DNA mutants for virulence on wheat and stress responses. **(A)** The five mutants were tested alongside the WT control isolate for their virulence on the leaves of a susceptible wheat cultivar. The mutants 4-124, 4-158, 5-51, and 15-120 exhibited reduced virulence when scored 21 days post-infection. In contrast, 9-70 exhibited WT levels of virulence. **(B)** The five strains were tested alongside the WT for sensitivity to stress on rich agar plates. Stress conditions were osmotic (sorbitol), oxidative (H_2_O_2_), and cell wall stress (Calcofluor White – CFW). The T-DNA mutants 4-124 and 4-158 exhibited hypersensitivity to oxidative and osmotic stress. **(C)** *In vitro* hyphal growth assays on tap water agar (TWA). Each strain was spotted onto TWA and incubated for 10 days to enable radial hyphal growth. Diminished hyphal growth rates were observed for strains 4-124, 4-158 and 15-120.

Each isolate was retested for virulence against the susceptible wheat cv. Riband, on three leaves, and symptom development on wheat was monitored for 21 days post-inoculation. Photos were taken 21 days post-inoculation (**Figure 1A**). Altogether, each isolate was tested in an attached wheat leaf screen at least four times with an IPO323 WT control to compare symptom progression. Four of the five mutant isolates tested had reproducibly reduced virulence on wheat leaves, manifesting as delayed symptom development restricted to only limited chlorosis by 21 dpi (**Figure 1A**). In contrast, isolate 9-70 (the ninth transformation round, picked colony 70) showed disease symptom development that was not significantly distinguishable from WT strains, going against the observations from an initial one-leaf phenotypic screen (Rudd, unpublished). The typical levels of disease symptoms for each strain are shown in **Figure 1A**. These assays confirmed previous work, suggesting mutant isolates 4-124, 4-158, 5-51, and 15-120 were reduced virulence mutants of *Z. tritici*.

### Further characterisation of the T-DNA mutants for *in vitro* growth and stress defects

Further phenotyping of the T-DNA mutant isolates *in vitro* was carried out using different fungal stressors, imparting osmotic, oxidative and cell wall stress, and for hyphal growth induction on tap water agar (TWA). For stress sensitivity testing, each isolate was tested a minimum of three times against each stress, and final images of fungal growth spotting plates were taken after five days. The typical results of these assays are shown in **Figure 1B**. Isolates 4-124 and 4-158 displayed hypersensitivity to both osmotic and oxidative stress, whilst 15-120, 5-51 and 9-70 appeared to tolerate all the fungal growth stress conditions tested.

To assess the ability of the five isolates to switch to hyphal growth each was also spotted onto TWA, with an IPO323 spot used as the WT control comparison (**Figure 1C**). Two of the five mutant isolates (5-51 and 9-70) in the hyphal growth assays showed a typical WT hyphal growth phenotype. In contrast, mutant isolate 15-120 displayed the most drastic reduced hyphal growth, compared to the other mutant isolates, with only a few short branches very close to the spot edge. A reduction was also seen for isolates 4-124 and 4-158, exhibiting a similar short, hyperbranched hyphal growth phenotype. This reduction paralleled a similar reduction in virulence and was also associated with hypersensitivity to oxidative and osmotic stress in both strains, suggesting potential functional links between these phenotypes.

### Detection of T-DNA insertion sites in the four T-DNA mutant isolates

Whole-genome re-sequencing was then performed on the four ATMT IPO323 mutants described. The initial objective was to determine the number and location of T-DNA insertions in the four strains with reduced virulence on wheat.

**Figure 2:**
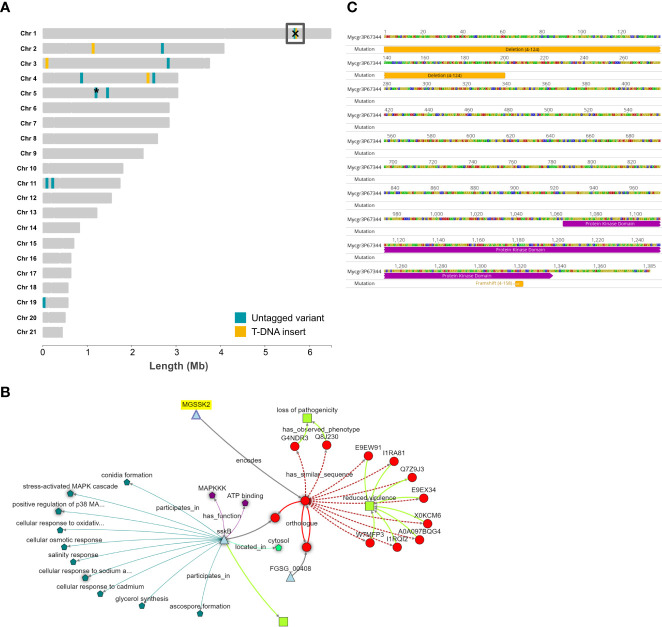
The “landscape” of genomic mutation events and the identification of a gene, SSK2, affected by mutations in two independent T-DNA strains. **(A)** The 21 chromosomes of the reference isolate IPO323 with the positions of tagged (T-DNA) and untagged (SNPs/indels) identified in the re-sequenced mutant strains. Generated using R package chromoMap (v. 0.3.1) (Anand & Rodriguez Lopez, 2022). Yellow shading indicates T-DNA insertion sites, blue shading represents untagged mutations. The box on chromosome 1 highlights a particular gene affected by both types of mutations in two independent mutant strains, 4-124 and 4-158. The asterisk on chromosome 5 represents that there are untagged variants in genes that are close neighbours, namely ZtritIPO323_04g09806 and ZtritIPO323_04g09809. **(B)** The gene commonly affected by the mutations, *SSK2*, in mutants 4-124 and 4-158, with the positions of the mutation events indicated. **(C)** Zymoseptoria KnetMiner knowledge network analysis indicating that *SSK2* is an orthologue of the corresponding yeast and fungal gene encoding a mitogen-activated kinase kinase kinase (MAPKKK).

Previous analysis of *Agrobacterium tumefaciens*-mediated transformation (ATMT) in fungi has demonstrated that a single T-DNA insertion event is the most frequent (Michielse et al., 2005). In agreement, only one T-DNA insert was detected in each strain for the four ATMT mutant isolates with reduced virulence analysed. **Table 1** shows the seven genes potentially affected by these integrations across all four strains. Of these seven, six genes were identified as potentially being affected by intergenic insertion, insertion occurring in close proximity to 5′-upstream regions or 3′-downstream regions of a gene(s). One gene was directly impacted by intragenic insertion of the T-DNA, that is insertion within a gene. **Figure 2A** shows the chromosomal locations of the genes affected by the T-DNA inserts and untagged variants. The figure emphasises that there was no obvious locational bias for the T-DNA integration events for the small number of events investigated.

**Table 1:** Genes affected by T-DNA insertion.

IPO323 Mutant Isolate	IPO323 Chromosome	T-DNA Insert Location	Putatively Impacted Genes	Interpro Name	Insert Location
**4-124**	1	5313974-5315085	ZtritIPO323_04g02061	Mitogen-activated protein (MAP) kinase kinase kinase Ssk2/Ssk22	Intragenic
			ZtritIPO323_04g02062	WD40-repeat-containing domain superfamily	Upstream
**4-158**	3	99207-99214	ZtritIPO323_04g07162	Gamma-glutamyl cyclotransferase-like	Downstream, 3’UTR
			ZtritIPO323_04g07163	Zn(2)-C6 fungal-type DNA-binding domain	Downstream, 3’UTR
**5-51**	2	1057013-1057051	ZtritIPO323_04g05837	Major facilitator superfamily	Downstream, 3’UTR
**15-120**	4	2261923-2261926	ZtritIPO323_04g09207	Molybdenum cofactor sulfurase, C-terminal;MOSC, N-terminal beta barrel	Downstream, 3’UTR
			ZtritIPO323_04g09208	Arf GTPase activating protein	Upstream

### A number of intragenic untagged SNP variants were also detected in the T-DNA insertion mutants

As previously discussed, “untagged” variations, including SNPs, insertions and deletions (indels), can occur in genomes and potentially be responsible for phenotypic changes. Our focus was on detecting intragenic variation that could most readily explain the mutant phenotypes. As a result, the full scope of intergenic variation has not been assessed. We next sought to determine the presence and number of additional intragenic untagged mutations in the mutant strains. These were labelled either isolate-specific, present in a single isolate only, or common, in two or more isolates. This analysis identified seventeen intragenic untagged variants, seven of which were single isolate specific. SNPeff identified four isolate-specific high-impact changes to proteins with predicted functions, two of which were in the 15-120 mutant. The genes were affected by three frameshifts, while one gained a stop codon mutation, as listed in **Table 2**. Three further moderate impact untagged mutations specific to isolate 15-120 were also identified (**Table 2**).

**Table 2:** Genes affected by unlinked mutations.

IPO323 Mutant Isolate	IPO323 Chromosome	Putatively Impacted Genes	Interpro Name	SNPeff Predicted Impact	Mutation
**4-124**	5	ZtritIPO323_04g09806	N/A	High	Frameshift^1^
	4	ZtritIPO323_04g08686	N/A	Moderate	Conservative in-frame deletion^2^
	5	ZtritIPO323_04g09929	P-type ATPase	Moderate	Missense variant^3^
	5	ZtritIPO323_04g09809	Alpha/beta hydrolase fold-3	Low	Synonymous variant^4^
**4-158**	1	ZtritIPO323_04g02061	Mitogen-activated protein (MAP) kinase kinase kinase Ssk2/Ssk22	High	Frameshift
	4	ZtritIPO323_04g08686	N/A	Moderate	Conservative in-frame deletion^2^
	5	ZtritIPO323_04g09809	Alpha/beta hydrolase fold-3	Low	Synonymous variant^3^
**5-51**	5	ZtritIPO323_04g09806	N/A	High	Frameshift^1^
	11	ZtritIPO323_04g03036	Zinc finger,GATA-type;PAS domain;PAC motif	High	Frameshift
	4	ZtritIPO323_04g08686	N/A	Moderate	Conservative in-frame deletion^2^
	5	ZtritIPO323_04g09929	P-type ATPase	Moderate	Missense variant^3^
	5	ZtritIPO323_04g09809	Alpha/beta hydrolase fold-3	Low	Synonymous variant^4^
**15-120**	2	ZtritIPO323_04g06395	Transglutaminase-like	High	Stop gained
	11	ZtritIPO323_04g02979	Pyridoxal phosphate-dependent transferase	High	Frameshift
	3	ZtritIPO323_04g08085	Piwi domain;PAZ domain; Argonaute, linker 1 domain	Moderate	Disruptive in-frame deletion
	4	ZtritIPO323_04g09248	ATPase, V0 complex, subunit e1/e2	Moderate	Missense variant
	19	ZtritIPO323_04g05318	N/A	Moderate	Missense variant

^1^Matching frameshift mutation; ^2^Matching in-frame deletion; ^3^Matching missense variants; ^4^Matching synonymous variants. N/A, not applicable (no InterPro name found).

No predicted high-impact untagged mutations were common to all four isolates (**Table 2**). However, the same “common” frameshift mutation was seen in two isolates, 4-124 and 5-51, in a predicted gene of unknown function (**Table 2**, ZtritIPO323_04g09806). Irrespective of this, the frameshift in this gene was considered unlikely to explain the phenotypes, supported by the fact that isolate 5-51 had a different hyphal growth phenotype compared to 4-124 (**Figure 1C**). There were two predicted moderate-impact SNP mutations in three mutant isolates and one common low-impact mutation (**Table 2**). These three variants were in the same position and were of the same type in mutants 4-124 and 5-51. Two of these same variants were also in the 4-158 mutant, but a moderate missense variant was absent (**Table 2**, ZtritIPO323_04g09929). Labelled with an asterisk in **Figure 2A** on chromosome 5 is a region where two common variants occurred in the same neighbourhood of genes. On **Table 2** these are ZtritIPO323_04g09806 and ZtritIPO323_04g09809.

Altogether this makes four common variants, one predicted high impact, two moderate and one low. The 4-124, 4-158, and 5-51 mutant isolates were generated using potentially the same parental IPO323 glycerol stock (strain names indicate transformation experiments four and five, followed by their respective colony numbers). The presence of these “common” untagged mutations could result from the random mutagenesis method causing similar mutations or, we consider more likely, to be present in the IPO323 stock used in the experiment prior to the mutagenesis. Regardless, we were confident that the “common” variants described in 4-124, 4-158, and 5-51 were unlikely to explain the differing hyphal growth properties.

There was one remarkable finding amongst the isolate-specific untagged high-impact variants which generate loss-of-function mutations in proteins. The previous analysis of T-DNA insertion sites in mutant 4-124 identified an intragenic insertion in the promoter and N-terminal region of a putative MAPKKK protein, orthologous to those encoded by the *SSK2/SSK22* genes in yeast (Maeda et al., 1995: protein ID YNR031C). While analysing the untagged mutations in strain 4-158, we identified a high-impact frameshift mutation in the same gene (Mycgr3G67344/ZtritIPO323_04g02061). Therefore, this gene is listed in **Tables 1** and **2** and highlighted with a box on chromosome 1 in **Figure 2A**. Incidentally, both 4-124 and 4-158 mutant isolates were shown to have reduced virulence and similar stress sensitivities and hyphal growth defects. These similar sensitivities indicated that there may have been a common genetic basis for the shared phenotypes of these two mutant strains, which was later uncovered by genome sequencing. **Figure 2B** highlights that the respective individual mutations occurred at opposite ends of the predicted protein, with the SNP frameshift occurring towards the C-terminal protein end but still within a conserved protein kinase sub-domain.

### KnetMiner analysis supports ZtSSK2 as an orthologue of yeast and fungal SSK2 MAPKKKs which function in one arm of the HOG1 MAPK pathway

Ahead of any functional complementation attempts and to seek out further information on the Zt*SSK2* candidate gene, we performed a KnetMiner analysis (Hassani-Pak et al., 2021) for predicted orthologues and any associated phenotypic information. The Zymoseptoria KnetMiner draws knowledge graphs from large biological databases and literature from a select few fungi, including *Saccharomyces* yeast, *Fusarium graminearum*, *Neurospora crassa* and *Aspergillus nidulans* (https://knetminer.com/Zymoseptoria_tritici/). **Figure 2C** shows the knowledge graph generated for Zt*SSK2* using its JGI identifier Mycgr3G67344. The *Z. tritici* gene (MGSSK2) is highlighted and linked to the protein it encodes. It is then further linked to orthologues and proteins with “similar sequences”. This analysis identified orthologues in *Fusarium graminearum* (NCBI:txid5518) and *Aspergillus nidulans* FGSC A4 (NCBI:txid227321). The database for the latter has more associated information on cellular activities and functions. For the similar sequences identified, the majority also share a similar predicted or validated phenotype, “reduced virulence”, which agrees well with the observed phenotype of the 4-124 and 4-158 mutants.

### Complementation with WT Zt*SSK2* restored full *in planta* disease symptoms, hyphal growth and WT stress responses to both 4-124 and 4-158

**Figure 3:**
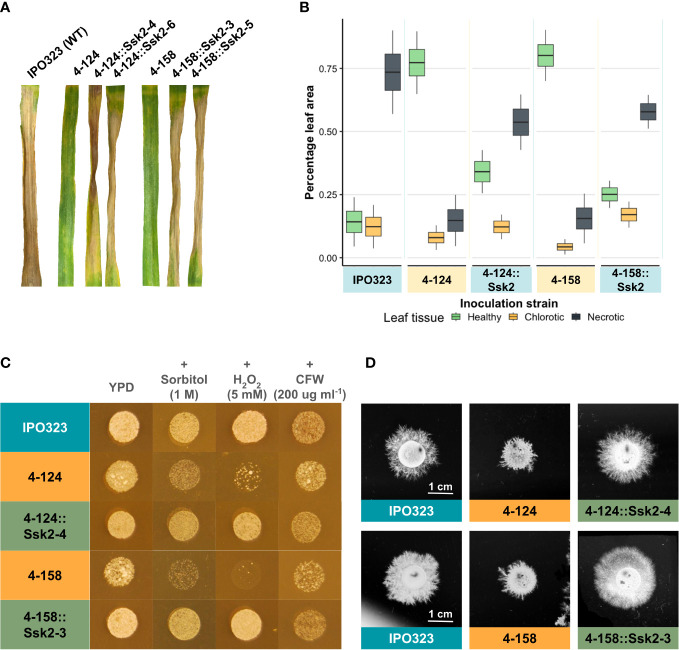
Re-introduction of the WT allele of SSK2 restores full virulence and stress responses to both the 4-124 and the 4-158 mutants. **(A)** Wheat leaf infection assays performed with the WT fungus, the two mutant isolates and both mutant isolates complemented with the WT *SSK2* gene (2 independent strains for each shown). The re-introduction of the functional *SSK2* gene restored virulence to both mutants. Photos were taken at 21 dpi. **(B)** Image-based (LemnaGrid) quantification of disease levels on the mutants and *SSK2* complemented strains on infected leaves. The percentage of infected tissue expressing no symptoms (healthy), chlorosis or necrosis is shown. **(C)** The *SSK2* complemented strains restored WT osmotic and oxidative stress sensitivity levels to the original 4-124 and 4-158 mutants. **(D)** The *SSK2* complemented strains generated WT levels of hyphal growth on TWA. Images were taken 10 days after inoculation.

We generated gene complementation constructs to determine whether the loss of function of ZtSSK2 was responsible for the reduced virulence and stress sensitivity of the 4-124 and 4-158 mutants. PCR products were obtained that included the native promoter, the complete coding sequence, and the terminator regions of the gene. These were cloned into vector pCGEN and used in *Agrobacterium-mediated* fungal transformation on the 4-124 and 4-158 mutants. The resulting transformants were then tested for increased ability to cause STB disease relative to the original mutants and compared to the WT strain. **Figure 3A** shows that multiple independent complemented strains from each parental background were restored for virulence on wheat, supporting the notion that the loss of SSK2 function in both the 4-124 and 4-158 strains was responsible for the reduction in virulence. **Figure 3B** provides quantitative data for the observed disease levels incorporating multiple replications.

The “High Osmolarity Glucose” HOG1 pathway is activated in fungi in response to multiple stresses. **Figure 1C** showed mutant isolates 4-124 and 4-158 have increased sensitivity to oxidative (hydrogen peroxide) and osmotic stress (sorbitol) during blastosporulation on rich nutrient agar. Using the original mutants, the WT strains, and the complemented strains, we next tested whether the re-introduction of functional SSK2 would restore normal sensitivity (reduced hypersensitivity) to these stressors. **Figure 3C** shows the results of these assays for a complemented strain (representative of three tested with identical results). The figure shows that the re-introduction of *SSK2* into both the 4-124 and 4-158 mutants resulted in a loss of the hypersensitivity to osmotic and oxidative stress seen in both original mutants. In contrast, there was no change in sensitivity to calcofluor white (CFW) which is commonly used to impose cell wall stress and is primarily thought to evoke activation of the cell wall integrity MAPK pathway mediated through SLT2 (Mehrabi et al., 2006a).

The other commonly shared phenotype in the Zt*SSK2* mutant strains was the aberrant hyphal growth exhibited on TWA. The two mutant strains consistently produced less extended filaments, which appeared to be hyper-branched compared to its parental strain, IPO323. **Figure 3D** shows this alongside the complementation results with a functional Zt*SSK2* after 10 dpi on TWA. The representative complementation (one of three tested with identical results) mutant shown displays the restored typical WT hyphal growth phenotype *in vitro*. **Figure 3D** shows that the re-introduction of *SSK2* fully restores hyphal growth to both the 4-124 and 4-158 mutants, again supporting that loss of this gene was responsible for the original phenotypes observed for both strains. The data also confirm a previously suggested link between reduced virulence phenotypes and aberrant growth morphologies for both strains.

### Fungicide sensitivity and kinase activity assays place ZtSSK2p upstream of ZtHOG1p and with non-redundant functionality to the MAPKKK, ZtSTE11

**Figure 4:**
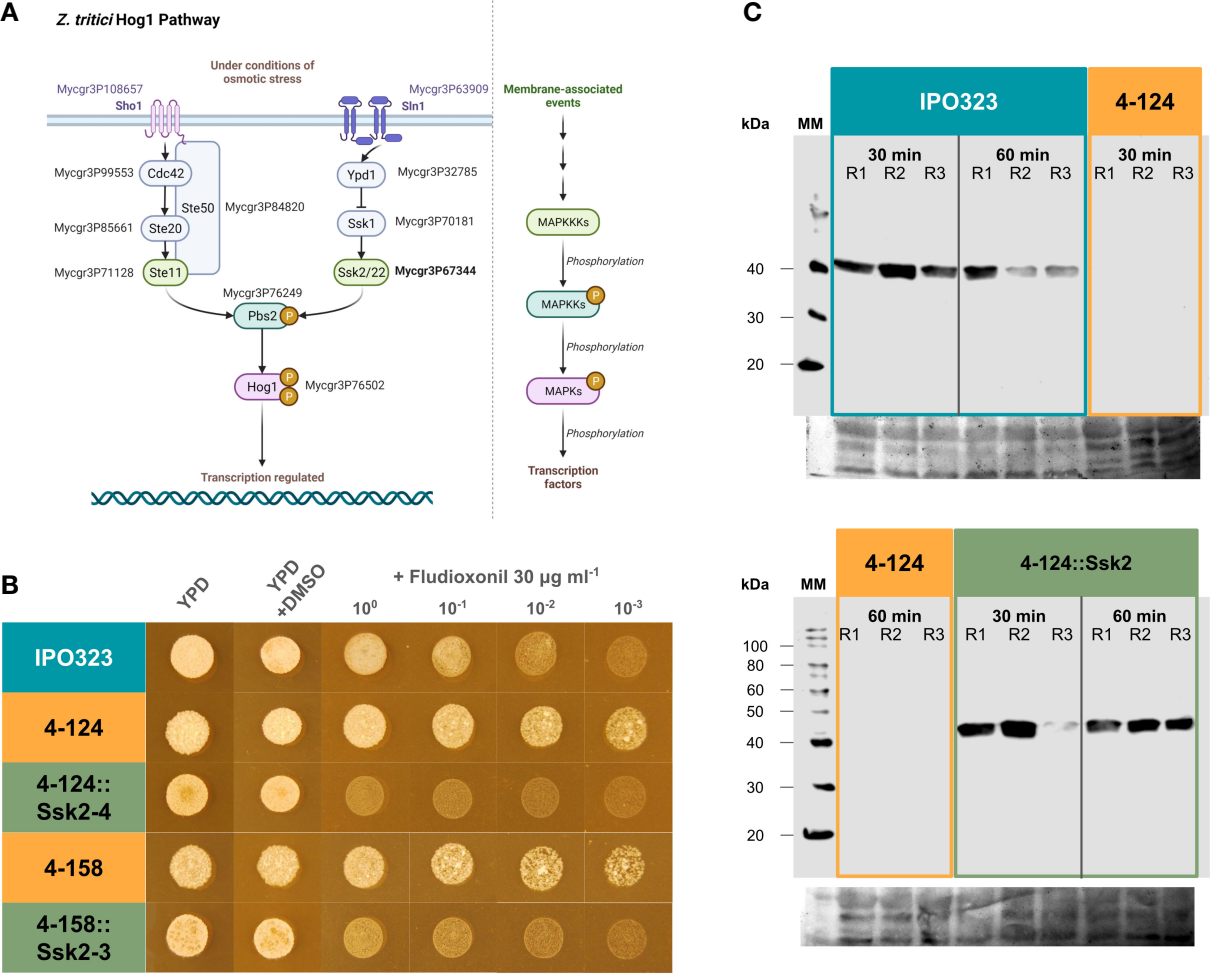
The SSK2 MAPKKK is an upstream activator of the HOG1 MAPK pathway and functions non-redundantly to activate the HOG1 protein under stress. **(A)** Representation of the HOG1 signalling pathways characterised in the yeast *Saccharomyces cerevisiae*. Note that HOG1 can be activated by two discrete arms involving either the STE20 or SSK2 MAPKKKs. The orthologous *Z. tritici* genes are labelled with the JGI protein identifiers; Mycgr3P108657 (Sho1), Mycgr3P63909 (Sln1), Mycgr3P99553 (Cdc42), Mycgr3P85661 (Ste20), Mycgr3P71128 (Ste11), Mycgr3P84820 (Ste50) Mycgr3P32785 (Ypd1), Mycgr3P70181 (Ssk1), Mycgr3P67344 (Ssk2), Mycgr3P76249 (Pbs2) and Mycgr3P76502 (HOG1). Created with BioRender.com. **(B)** Fludioxonil sensitivity assays indicate that *SSK2* is an upstream activator of HOG1. HOG1 mutants are less sensitive to this fungicide. The 4-124 and 4-158 strains had a similar reduced sensitivity. However, sensitivity was restored in the complemented strains. The spots indicate serial dilutions of spores. **(C)** HOG1 protein activity assays demonstrate that SSK2 is solely responsible for activating HOG1 in response to oxidative stress. Western blotting using anti-p38 MAPK antibodies to report on the activation of fungal HOG1 proteins. This protein displayed no activity without SSK2p, which was restored in the complemented strains. R1-R3 = replicated experiments. The fungicide sensitivity data and the HOG1 kinase activity data suggest that SSK2p has non-redundant functionality with the STE20 MAPKKK in activating HOG1 under specific conditions.

Based on knowledge of Saccharomyces yeasts and other fungi, *SSK2* is likely to function as one of two MAPKKKs that can activate the HOG1 MAPK in response to specific cues (Cheetham et al., 2007; Brewster & Gustin, 2014). **Figure 4A** shows a representation of the HOG1 signalling pathways. Many orthologous genes in these pathways have been identified in the *Z. tritici* genome, and the MAPKKK STE11 and HOG1 are required for full virulence (Mehrabi et al., 2006b; Kramer et al., 2009). Two previous studies on Zt*SSK1* (upstream of *SSK2*) and Zt*HOG1* mutants have also demonstrated increased resistance to a class of fungicides in mutants, as has also been seen for orthologues in other fungal pathogens (Mehrabi et al., 2006b; Yemelin et al., 2017). To place the predicted *SSK2* in the HOG1 pathway, we tested whether the original mutants exhibited this increased sensitivity and whether sensitivity was restored by complementation with *SSK2*. To this end, we performed a fungicide sensitivity *in vitro* growth assay. This assay revealed that Zt*SSK2* disrupted mutants 4-124 and 4-158 displayed increased resistance to the phenylpyrrole fungicide fludioxonil, exhibiting similar enhanced growth phenotypes at higher concentrations (**Figure 4B**). This observation supports previous reports by Mehrabi et al. (2006b) and Yemelin et al. (2017) on HOG1 and SSK1, respectively. Unlike the disrupted mutant isolates, the IPO323 reference strain and *Δ*Zt*SSK2*::Zt*SSK2* complementation strain on YPD supplemented with 30 μg ml^-1^ fludioxonil were more sensitive to fludioxonil. Therefore, these data are consistent with the idea that ZtSSK2p also acts upstream of ZtHOG1p and contributes to fungicide sensitivity independent of the other MAPKKK, STE11.

Much of the previous data has implied that the SSK2 MAP3K functions upstream of the HOG1 MAPK in one of the two potential arms leading to HOG1 kinase activation. To test this link more directly, we studied the activation of the HOG1 protein using anti-active MAPK antibodies and western blotting. Activated MAPK proteins can be detected using anti-active antibodies, which only cross-react with MAPKs when dual phosphorylated in the activation loop region. This dual phosphorylation is performed by the upstream MAPKK, activated by phosphorylation through MAPKKKs. We performed western blots using MAPK p38 anti-active antibodies, which recognise the mammalian ortholog of the fungal HOG1. As such, (anti-rabbit) p38 MAPK antibodies can also be used to test for the activation of HOG1 in fungi (Han et al., 1994). Western blotting was done on protein extracts from 4-124, 4-124::SSK2 complemented strains and IPO323 under oxidative stress induced by H_2_O_2_. **Figure 4C** shows that the activation (phosphorylation) of HOG1 was abolished entirely in the ZtSSK2 mutant (4-124), whereas the WT IPO323 and representative *Δ*Zt*SSK2*::Zt*SSK2* complementation strain isolate displayed strong activation. These data provide direct supporting evidence that HOG1 activation, through its dual phosphorylation, is an event downstream and requires the function of the SSK2 MAPKKK in response to oxidative stress. The data suggests, like the fungicide data, that the ZtSTE11 MAPKKK cannot compensate for the loss of function of ZtSSK2 in this response.

### Analysis of early *ZtSSK2*-mediated fungal gene expression on wheat leaves by global RNA-sequencing

To probe the consequence of loss of SSK2 function on infection-related gene expression, we analysed the *in planta* transcriptome of the 4-124 mutant isolate through RNA-sequencing. Samples were taken at two early *in planta* infection time points, 6 h and 24 h. These span the normal early switch to hyphal growth seen in the WT strain (including first stomatal penetration events), used as a control to compare overall gene expression across this period. Unfortunately, due to a technical error in RNA sequencing, one replicate from the 6 h IPO323 samples was unusable in the analyses. Another important initial observation was that the “WT” IPO323 strain appeared to have lost a small accessory chromosome, chromosome eighteen. This loss was flagged by the high number of differentially expressed (DE) genes mapping to the chromosome, followed by discovering the lack of reads mapping to any gene on this chromosome in the WT strain. This dispensable chromosome is highly prone to loss during strain cultivation (Kellner et al., 2014; Möller et al., 2018) but has no known impact on virulence or any other associated phenotype. Therefore, all reads mapped to chromosome eighteen in the SSK2 mutant were removed from subsequent analyses to identify DE genes elsewhere in the genome.

**Figure 5:**
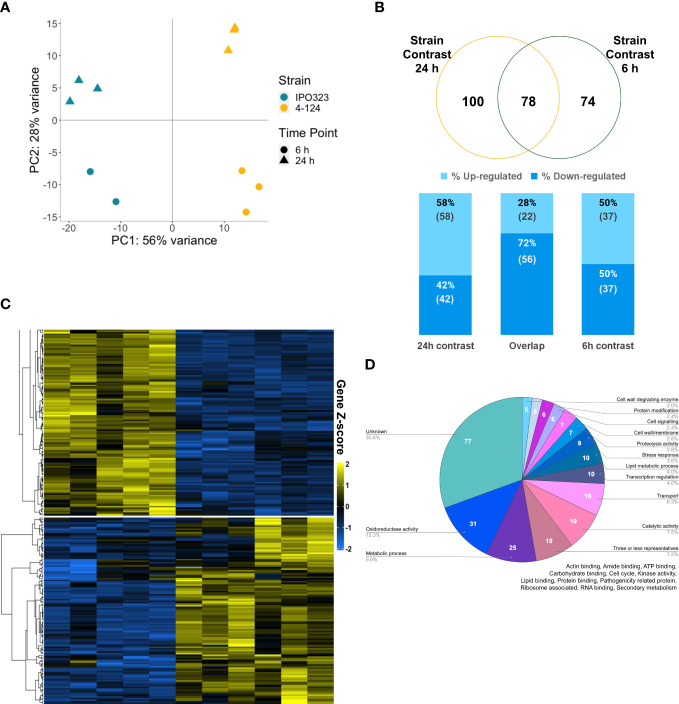
The SSK2 MAPKKK affects the fungal transcriptome during the early colonisation of wheat leaves. **(A)** Principal component analysis (PCA) plot of sample distances based on vsd transformed gene count data of the eleven sequenced RNA samples mapped to the *Z. tritici* transcriptome. PC1 explains 56% of the sample variance; this splits IPO323 (blue) and 4-124 (yellow) RNA samples. PC2 explains 28% of the variance, and this splits RNA samples by time, 6 h (triangle) and 24 h (spot). **(B)** Venn diagram showing the number of differentially expressed genes (DEGs) shared and unique to these two contrasts of 4-124 versus IPO323 at 24 h and 6 h timepoints. Stacked percentage bar chart showing the percentage of genes up-regulated (lighter blue) versus down-regulated (darker blue) in the mutant versus WT. These include those which contrast individually at 24 h,6 h and overlapping DEGs in “Both contrasts”. Numbers in brackets below the percentage are the actual number of DEGs. **(C)** Expression patterns of the differentially expressed genes using a heatmap generated with “vsd” normalised and log-transformed count data and clustered using PAM (partition around medoids). Gene Z-score scale represents the number of standard deviations away from the mean, averaging across the samples. Cluster 1 broadly represents the genes down-regulated in the 4-124 mutant (yellow bar) vs *Z. tritici* IPO323 (blue bar), and Cluster 2 represents up-regulated genes in the mutant versus the IPO323—generated using DeSeq2 and ComplexHeatmaps in R studio. **(D)** Classification of genes into 44 broad predicted functional groups. Expressed genes annotated through GO and Joint Genome Institute data, combined with results retrieved from BLASTp, InterProScan and Blast2GO analyses.

**Figure 5A**, depicting a principal component analysis (PCA), shows that the biological replicates cluster together (circled) and that principal components split the samples by strain and timepoint. This plot indicates that the mutant isolate 4-124 and WT IPO323 expression patterns differed more by strain than by sampling time. Gene expression levels were then individually compared between the *Z. tritici* mutant 4-124 and IPO323 strain at each time point. Genes were considered “differentially expressed” at a cut-off where the average expression was affected by a factor of 1-log2 fold (equates to 2-fold change in expression), and false discovery rate adjusted p-values were less than or equal to 0.05. However, due to the failed run of a biological replicate in IPO323 at 6 h, the WT expression is based on only two replicates, increasing the chance of false positives and negatives. As a result, for this analysis, more emphasis was placed on investigating differentially expressed genes (DEGs) identified in the strain contrast (4-124 vs WT) at 24 h, where 3 biological replicates were available for both mutant and WT strain.

In total, 252 significantly differentially expressed genes (DEGs) were identified, listed in **Supplementary Table S1**. This list included 100 genes from the 4-124 vs IPO323 strain comparison at 24 h, and 74 DEGs at 6 h. **Figure 5B** shows that an additional 78 genes overlap between the different comparisons. This overlap indicates that similar biological responses occur at both timepoints, most probably due to the relatively close sampling time points (6 h and 24 h). **Figure 5B** also shows the percentage of up/down-regulated genes in 4-124 versus IPO323. From this, we can see that more genes were downregulated in the Zt*SSK2* mutant than IPO323, with 135 unique DEGs across the three comparisons versus 117 up-regulated. Two clusters of genes were identified through PAM K-medoids testing, as shown in **Figure 5C**. Genes in cluster one are up-regulated in IPO323 compared to 4-124, whilst genes in cluster two show the opposite pattern.

Classification of DEGs into their predicted functions identified 44 functional groups, annotated through GO and Joint Genome Institute data, combined with results retrieved from BLASTp, InterProScan and Blast2GO analyses. This was done similarly to Yemelin et al. (2021), who reported a recent *in vitro* growth transcriptome analysis on mutant isolates, which included a ΔZtHOG1 mutant isolate (the downstream MAPK activated by the MAPKKK Zt*SSK2*). As shown in **Figure 5D**, 175 genes (69.4%) across both 6 h and 24 h strain contrasts could be classified into functional categories. For ease of representation, the fifteen largest categories are shown. For functional groups with three or less differentially expressed members, these were merged into a “Three or less representatives” category (full list of DEGs shown in **Supplementary Table S1**).

To determine whether any functional category was overrepresented in the RNAseq data, we performed a GO enrichment analysis using OmicsBox (v.1.3.11). Fisher’s Exact test identified one over-represented GO term that met the false discovery rate (FDR) p-value <0.05, which was “oxidoreductase activity” (GO:0016491, FDR p = 4.75E^−2^). **Supplementary Table S1** provides all data for the fungal DEGs and shows the numbers of genes in the functional categories in **Figure 5D** and for the clusters identified in the heatmap in **Figure 5C**. Interestingly, the total number of “Stress response” associated genes was lower in cluster two, where expression is up-regulated for the mutant. This may again indicate the reduced activation of HOG1 in 4-124 has downstream for the expression of genes involved in stress responses.

Finally, we sought to identify any expression changes for any known HOG1 transcriptionally regulated genes, bringing in literature from other fungi and yeasts. For this, we again used the Zymoseptoria KnetMiner tool (https://knetminer.com/Zymoseptoria_tritici/). The KnetMiner keyword search function was used to identify a list of candidate genes putatively associated with HOG1, response to various stresses and developmental stage associated genes from model fungi and the yeast *S. cerevisiae*. These search terms “HOG1”, “oxidative stress”, “osmotic stress”, “cell wall integrity”, “hyphal growth”, “filamentous growth”, and “dimorphism” generated a list of 1964 genes purportedly linked. A total of 29 of these genes were identified as DE within the 24 h strain comparison (**Supplementary Table S1**). Matching the same search terms to Yemelin et al. (2021) *in vitro* RNAseq delta HOG1 mutant data identified 47 total genes linked, 6 of which are also within the 24 h strain comparison presented in this study (**Supplementary Table S1**). These represent strong candidates for SSK2-HOG1 regulation. **Table 3** displays the combined results of these analyses from our study and highlights several specific proteins which may confer oxidative and other stress responses through activated ZtSSK2 and HOG1 in *Z. tritici*.

**Table 3:** Summary of KnetMiner searches.

*Z. tritici* differentially expressed genes 24 h			KnetMiner Search Match (Y/N)						
Gene Name	Description	Log2 FC	HOG1	Oxidative stress	Osmotic stress	Cell wall integrity	Hyphal growth	Filamentous growth	Dimorphism
ZtritIPO323_04g10603	Linoleate diol synthase	−2.59	N	Y	N	N	N	N	N
ZtritIPO323_04g11352	Glucan 1,3-beta-glucosidase	−2.05	N	N	N	Y	N	N	N
ZtritIPO323_04g02810	Siderophore-dependent iron transporter	1.87	N	N	N	N	Y	N	N
ZtritIPO323_04g00700	THI5-LIKE	−2.04	N	Y	N	N	Y	N	N
ZtritIPO323_04g00819	NADP-dependent alcohol dehydrogenase like	−2.72	Y	Y	N	N	N	N	N
ZtritIPO323_04g01183	Monomeric glyoxalase I	−2.26	Y	Y	Y	N	N	N	N
ZtritIPO323_04g01615	Basic-leucine zipper domain	3.29	N	Y	N	N	N	N	N
ZtritIPO323_04g07726	Related to Woronin body major	1.99	N	Y	N	N	N	N	N
ZtritIPO323_04g10309	Glycoside hydrolase family 17	2.08	Y	Y	N	Y	N	N	N
ZtritIPO323_04g02407	Aryl-alcohol dehydrogenase Aad14	−2.69	N	Y	N	N	N	N	N
ZtritIPO323_04g02517	Glutathione-s-transferase like	−2.43	N	Y	N	N	N	N	N
ZtritIPO323_04g01836	Zinc finger transcription factor ace1 like	2.4	N	N	Y	N	N	N	N
ZtritIPO323_04g11895	MDR multidrug transporter	−3.04	N	Y	N	N	N	N	N
ZtritIPO323_04g13735	G-coupled receptor	−1.79	Y	Y	Y	Y	Y	Y	Y
ZtritIPO323_04g05630	Peptidase M3A/M3B catalytic domain	3.07	Y	Y	N	Y	N	N	N
ZtritIPO323_04g10608	Carboxylesterase B family	−2.43	N	Y	N	N	N	N	N
ZtritIPO323_04g08711	RNA binding	−2.26	N	Y	N	N	N	N	N
ZtritIPO323_04g09472	Lysophospholipase 2	3.37	N	Y	N	Y	Y	N	N
ZtritIPO323_04g02648	Phospholipase D/Transphosphatidylase	−2.59	N	N	N	N	Y	N	Y
ZtritIPO323_04g11589	Alcohol dehydrogenase -like domain-containing	−3.5	Y	Y	N	N	N	N	N
ZtritIPO323_04g00114	NADP-dependent mannitol dehydrogenase	−2.47	N	Y	N	N	N	N	N
ZtritIPO323_04g06038	2,3-butanediol dehydrogenase like	3.9	N	Y	N	N	N	N	N
ZtritIPO323_04g07298	Acid trehalase	−3.2	Y	Y	N	Y	N	N	Y
ZtritIPO323_04g08522	Glutamate decarboxylase	−2.92	Y	Y	N	N	N	N	N
ZtritIPO323_04g10685	Alternative oxidase	3.53	N	Y	N	N	Y	N	N
ZtritIPO323_04g12868	ABC transporter	2.87	Y	Y	N	Y	N	N	N
ZtritIPO323_04g08156	CAT1 catalase	−2.51	Y	Y	Y	Y	Y	N	N
ZtritIPO323_04g10208	Zinc-binding alcohol dehydrogenase like	−3.19	N	Y	N	N	N	N	N
ZtritIPO323_04g13370	3-ketoacyl-ACP reductase like	−2.93	N	Y	N	N	N	N	N
ZtritIPO323_04g03984	Lipase 3	4.15	N	Y	N	N	N	N	N
ZtritIPO323_04g04239	Helicase superfamily 1/2	3.25	N	Y	N	Y	Y	N	N
ZtritIPO323_04g04241	Acetate transporter GPR1/FUN34/SatP family	4.75	N	Y	N	N	N	N	N

#### Zt*SSK2* mutants still undergo similar early developmental changes on the leaf surface and do not distinctly affect the wheat transcriptome

**Figure 6:**
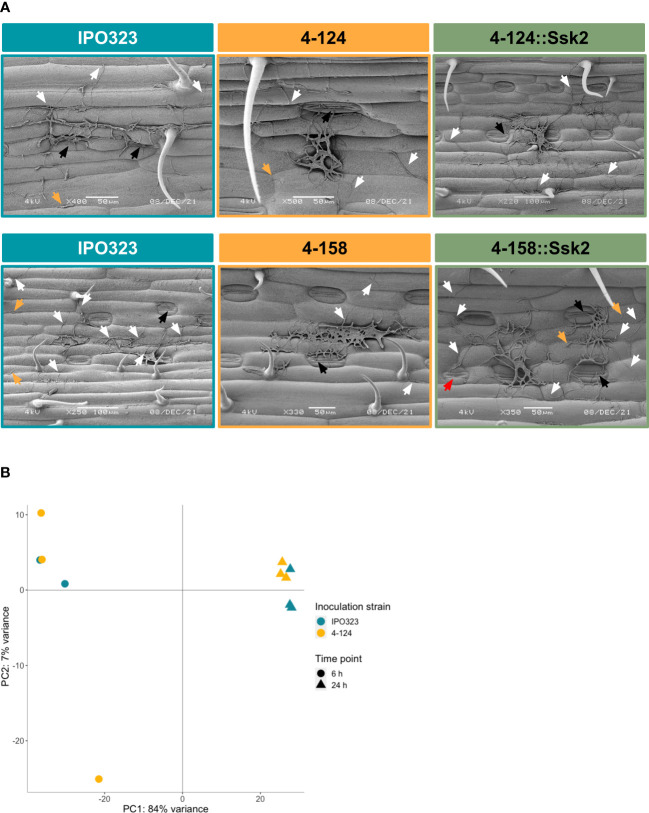
Microscopy and gene expression analyses suggest that wheat does not differentially recognise fungal SSK2 mutants from WT during early leaf infection. **(A)** SEM images were taken nine days post-inoculation of WT and SSK2 mutants on the wheat leaf surface. This analysis failed to identify any consistent differences between the tested strains suggesting that early developmental changes are not profoundly affected by the loss of SSK2 function during this initial infection stage. White arrows indicate spore germination, with ungerminated spores labelled by yellow arrows and attempts to penetrate stomata indicated by black arrows. In the representative image for the complemented 4-158 mutant, a red arrow indicates a filament extending over a closed stoma. **(B)** Principal component analysis (PCA) plot of sample distances based on vsd transformed gene count data of the eleven sequenced RNA samples mapped to the Chinese Spring wheat genome assembly. PC1 explains 84% of the sample variance; this splits the RNA samples by time, 6 h (triangle) and 24 h (spot). Only 7% of the variance is explained by PC2 (fungal strain). Overall, these data suggest that wheat is not differing in its response to early infection attempts by the WT and SSK2 mutant fungi.

**Figures 1C**, **3D** indicated that both the T-DNA *SSK2* mutants were affected for hyphal growth on water agar, when measured at later timepoints after agar inoculation. It was, therefore, possible that this defect may also be responsible for the failure of the mutants to cause full disease on wheat leaves. To test this, we inoculated wheat leaves with spores of mutants and WT strains and analysed cells qualitatively for surface germination at 1, 3 and 9 days post-inoculation using SEM. Qualitative assessments were only made due to simplicity and the fact that if stable genetic ablation of *SSK2* function arrested initial yeast to hyphal growth, this should be apparent in all cells analysed compared to the WT. **Figure 6A** shows representative images from 9 dpi. Overall, this analysis revealed no major differences in the frequency or level of germination, indicating that the initial germination events are similar in both mutants and WT. The figure also shows that some stomatal penetration events occur and that some hyphae grew across stomates without penetration. This analysis suggests that any hyphal growth (or other) infection-related morphological defects in these Zt*SSK2* mutants likely occurs later in the infection process and not at the initial point of spore germination on leaves.

Similarly, analysis of the wheat transcriptome data suggested that the host does not detect a significant early change in the infection biology between the WT and *SSK2* mutant strains of the pathogen (**Figure 6B** and **Supplementary Table S2**). Although some genes were differentially expressed, many lacked functional annotation, and there was no clear change in the expression of pathogenesis-related (or other gene categories) in the gene lists. The overall similarity of the host transcriptome response was emphasised in the PCA analysis (**Figure 6B**), which demonstrated samples (and replicates) separated on time, not by fungal strain.

## Discussion

### Whole-genome re-sequencing confirms the genetic instability of the *Z tritici* genome

Our analysis of the small set of random ATMT mutants demonstrated that only single T-DNA inserts in each isolate were observed. It has previously been shown that, on average, ATMT integrates only one copy of a T-DNA insert into the host genome, reducing the analyses’ complexity as there should be fewer functionally impacted genes (Michielse et al., 2005). This consistency also reduces the chances of multiple affected genes contributing to an observed phenotype. However, as shown here, the method (or other handling/storage steps) may also introduce “untagged” genomic variation. These are sequence changes not associated with the T-DNA integration event. In addition, T-DNA integrations have also been associated with other chromosomal aberrations, including rearrangements, deletion, duplication, and inversion (Weld et al., 2006).

Nevertheless, with the random ATMT collection, only a few common untagged variants were observed. The small numbers suggest that the parental strain had changed from the original sequence at a low level. A few putative mechanisms explaining how “untagged” variants arise during the transformation process have been suggested, including that the procedure itself is innately mutagenic; ergo, the stress applied to the genome by the transformation, selection, and regeneration process likely generates untagged effects (Kahmann & Basse, 1999; Maier & Schäfer, 1999; Mullins and Kang, 2001). The absence of chromosome 18 from the IPO323 version used in RNAseq is another extreme example of the high genome instability recognised for this fungus (Möller et al., 2018).

### The power of casting a genome-wide net for untagged variants in the mutagenomics approach

*Z. tritici* was the first genome of a filamentous fungus to have a fully sequenced, all chromosomes from telomere to telomere, reference isolate genome (IPO323) (Goodwin et al., 2011). This sequence has been an excellent scaffold for re-sequencing projects like the mutagenomics analyses described here. Current knowledge of the genome has built on the earlier studies that identified the presence of dispensable/accessory chromosomes, chromosome length, and number polymorphisms (McDonald & Martinez, 1991; Zhan et al., 2002; Wittenberg et al., 2009; Testa et al., 2015). However, next-generation sequencing technologies, and a reduction in the cost of using those technologies, have enabled an approach that has more power to identify changes that would have previously gone unseen. In addition, multiple studies have highlighted the diversity and instability of some areas of *Z. tritici* chromosomes (McDonald et al., 2016; Habig et al., 2017). Whilst the variation in mutant isolates detected here is relatively small compared to natural variation between strains in field populations, the fact it occurs points to significant genome instability in this organism, which may also contribute to its rapid evolution rate.

Identifying tagged integration events has traditionally used selective techniques for laboratory-generated mutants, including chromosome/primer walking, plasmid rescue and PCR-based methods. These have also been used successfully to identify T-DNA insertion and REMI integration sites in other fungi (Michielse et al., 2005; Urban et al., 2015). Whole-genome sequencing also enables the identification of other “untagged” mutations too. To add to the power of this approach in future, the parental strains used in the mutagenesis experiment should also have their genomes re-sequenced at the outset. This inclusion would help identify putative untagged effects as either “background” mutations in isolate stocks from those potentially commonly induced mutations due to the mutagenesis experiment. The fact that the reduced virulence and stress phenotypes in strain 4-158 were shown to be caused by a SNP mutation in *SSK2*, which was independent of the integration of the T-DNA, emphasises the importance of using whole genome re-sequencing approaches. How this mutation occurred remains currently unclear.

There also remain mutant strains from this analysis which require further characterisation, namely 15-120 and 5-51. Previous attempts to link candidates associated with intergenic T-DNA insertion sites in 5-51 and 15-120 (**Table 1**) to their respective phenotypes (**Figure 1**) were unsuccessful. However, after checking their whole genomes for untagged mutations the other likely candidates uncovered in this study (**Table 2**), highlighting the power of this mutagenomic approach. In the case of 5-51, a frameshift mutation in ZtritIPO323_04g03036 (Mycgr3G76651.1), the White Collar-1 (Zt*WCO1*) gene, we consider the most likely candidate given the recent literature on the importance this gene in dimorphic switching and the role of light-responsive proteins in orchestrating the *Z. tritici* infection (Kilaru et al., 2022). We considered the intergenic T-DNA insertion (**Table 1**) in 5-51 unlikely to explain the phenotype, and the other untagged variants (**Table 2**) are common to 4-124 which has a different phenotype (**Figure 1**). However, we recognise there could be other undetected chromosomal (or other) rearrangements that could also impart phenotypic changes in these strains.

### Efficient stress response pathways are interlinked with fungal virulence and hyphal growth traits

Fungi continuously sense and respond to environmental cues; molecular and genetic circuitry behind those mechanisms make “decisions” that affect the transitions into lifecycle phases (Lengeler et al., 2000). Therefore, the accuracy of detection and efficiency of those circuits is paramount for an organism’s survival. For example, the invading *Z. tritici* hyphae are exposed to the leaf apoplast environment of the wheat host. Keon et al. (2007) reported that this is a low-nutrient environment, and upon detection by the host, the fungus is exposed to several stressors. Reactive oxygen species, ions, plant signalling hormones and enzymes attacking major fungal cell wall components, and other microorganisms are just some examples of elements that an apoplastic fungal pathogen must “overcome” to cause infection (Rodriguez-Moreno et al., 2018). Fungal enzymes with oxidoreductase activity are involved in various cellular processes and have been linked to pathogenicity and protection against host-defence responses (Singh et al., 2021). Previous transcriptomic studies in *Z. tritici* identified a subset of oxidoreductases, namely secreted chloroperoxidases, which displayed up-regulation at 1 dpi peaking later at 4 dpi (Rudd et al., 2015). Similarly, in Palma-Guerrero et al. (2016), oxidoreductases were identified as the most up-regulated category at seven dpi of WT infection. Furthermore, oxidoreductases have previously been shown to be up-regulated in response to UV radiation to reduce the levels of free radicals (McCorison & Goodwin, 2020).

The HOG1 pathway has been described as a conserved and prolific regulator of fungal growth, involved in multiple stress responses, cell cycle progression, morphogenesis, cell wall biogenesis and virulence (Hohmann, 2009; Brewster & Gustin, 2014). Despite this, the control, upstream sensors, activation, and functions of the orthologous HOG1 pathways vary between fungi. The RNA sequencing results highlighted that the between the Zt*SSK2* mutant and WT *in planta* the largest differentially expressed category were genes with the GO term relating to oxidoreductase activity. Combined with the Zt*SSK2* mutants displayed enhanced sensitivity to oxidative stresses, it appears likely that the MAPKKK regulates the HOG1 pathway to improve resilience to this stress through regulating oxidoreductase activities. The western blot result indicated that in the Zt*SSK2* mutant, the activation of the HOG1 kinase is impacted under oxidative stress conditions. Whilst the lack of activation of HOG1 observed in the SSK2 mutant could also have been either delayed or extremely short-lived, the data presented suggest that the MAPKKK, ZtSTE11 cannot compensate for the loss of SSK2 function in responses to oxidative stress, fungicides or in virulence. In Kramer et al. (2009), it was noted that *ZtSTE11* null mutants were impacted in their virulence but were not sensitive to the fungal growth stressors tested, including oxidative stress using H_2_O_2_. Together these data suggest that SSK2p functions as the major arm in the HOG1 activation pathway in response to oxidative stress. The fact that both the MAPKKKs STE11 and SSK2 are independently required for full virulence on wheat suggests they may both be responsible for full (maximal) HOG1 activation to achieve this or that either or both proteins can affect virulence independent of HOG1. Double mutants (should they be viable) may be needed to test these ideas. However, what we report here parallels work from the animal pathogen *Candida albicans* which demonstrated that only SSK2 was able to activate HOG1 (Cheetham et al., 2007). Therefore, it may be that the STE11 MAPKKK may not function through the HOG1 MAPK in *Z. tritici*.

### The SSK2-HOG1 pathway is important for infection-related stress gene expression

Much remains to be understood about the pre-symptomatic latent phase of *Z. tritici* infection, particularly in the early pre-stomatal penetration period. However, evidence exists that this provides minimal external nutrition to early hyphal growth (Rudd et al., 2015; Palma-Guerrero et al., 2016). Previous transcriptomic studies *in planta* typically sample at 24h dpi as the earliest timepoint, to capture surface germination and early penetration of the leaf surface (Rudd et al., 2015). However, a consideration brought to attention by Fantozzi et al. (2021) is the asynchronous development of *Z. tritici in planta*. In short, invading hyphae follow their individual asynchronous developmental program, and expression profiles likely reflect this. However, our data showed a clear separation of overall transcriptomic data between the WT and mutant fungus, indicating that *SSK2* gene loss was a key driver for this, and was still exerting effects on the fungal transcriptome irrespective of any asynchronous development.

Before this work, the earliest time point for dual transcriptomic studies *in planta* was reported by Benbow et al. (2020). This study aimed to determine the plant responses to *Z. tritici* in two wheat cultivars, a resistant cultivar (cv. Stigg) and a susceptible (cv. Longbow), at four timepoints, starting at 6 h. They focused on the differing stress responses mounted by wheat against the fungus and identifying disease response genes involved in the early response to *Z. tritici*. As such, they did not detail any differences in the fungal transcriptome between cultivars. Whilst Benbow et al. (2020) saw a difference between host responses to infection in susceptible versus resistant cultivars, we show that there is a limited difference between the expression profiles of a susceptible cultivar responding to a reduced virulence mutant compared to a wild-type virulent strain, particularly at this early time point. This is supported by the observation that only a few germinated spore filaments were seen to have penetrated.

The leaf surface SEM imaging indicated little appreciable visual difference between the WT and defective Zt*SSK2* mutant strains, despite the noticeable phenotypic differences seen macroscopically *in vitro*. So, unless there is a significant difference in the surface PAMPs presented by the WT and mutant strains, it is difficult to envisage how the host would respond differently at such early stages. This could, however, change for later stages. It is conceivable that the reduced virulence of *SSK2* mutants could arise from a combination of reduced hyphal growth at later stages and greater sensitivity to some of the plant-imposed stresses discussed. Therefore, the exact phase of the infection cycle which imposes the most significant stress on the defective *SSK2* mutant, hindering its virulence, awaits further resolution. Despite this, the strains and tools developed in this initial “mutagenomic” analysis pave the way for an accelerated understanding of the genes and processes required for wheat infection by *Z. tritici*; more broadly, this offers a pipeline which could be exploited to understand the pathogenesis of fungal plant pathogens in general further.

## Data availability statement

The datasets presented in this study can be found in onlinerepositories. The names of the repository/repositories and accession number(s) can be found below: https://www.ncbi.nlm.nih.gov/, GSE225623, SAMN33272753, SAMN33272754, SAMN33272755, SAMN33272756.

## Author contributions

Undertaking the research – HB. Analysing data RK, DS, HB, JR, TA. Provided materials – CB, HW, EV. Writing manuscript – JR, HB, DS. Funding – RR, JR, KK. All authors contributed to the article and approved the submitted version.

## Supplementary material

Supplementary Table 1 The differentially expressed fungal genes (WT vs SSK2 mutant) at 6 and 24 hpi on the wheat leaf surface (.xls).

Supplementary Table 2 The differentially expressed wheat genes following early infection by WT and SSK2 mutant strains (.xls).

## References

[B1] Anand L. Rodriguez Lopez C. M. (2022). ChromoMap: an R package for interactive visualization of multi-omics data and annotation of chromosomes. BMC Bioinf. 23 (1), 33. doi: 10.1186/s12859-021-04556-z PMC8753883 35016614

[B2] Benbow H. R. Brennan C. J. Zhou B. Christodoulou T. Berry S. Uauy C.. (2020). Insights into the resistance of a synthetically-derived wheat to Septoria tritici blotch disease: Less is more. BMC Plant Biol. 20 (1), 1–23. doi: 10.1186/s12870-020-02612-z 32883202 PMC7469286

[B3] Benjamini Y. Hochberg Y. (1995). Controlling the false discovery rate: A practical and powerful approach to multiple testing. J. R. Stat. Society Ser. B. (Methodological) 57 (1), 289–300. doi: 10.1111/j.2517-6161.1995.tb02031.x

[B4] Bolger A. M. Lohse M. Usadel B. (2014). Trimmomatic: a flexible trimmer for illumina sequence data. Bioinformatics 30 (15), 2114–2120. doi: 10.1093/bioinformatics/btu170 24695404 PMC4103590

[B5] Brewster J. L. Gustin M. C. (2014). Hog1: 20 years of discovery and impact. Sci. Signaling 7 (343). doi: 10.1126/scisignal.2005458 25227612

[B6] Cheetham J. Smith D. A. da Silva Dantas A. Doris K. S. Patterson M. J. Bruce C. R.. (2007). A single MAPKKK regulates the Hog1 MAPK pathway in the pathogenic fungus *Candida albicans* . Mol. Biol. Cell 18 (11), 4603–4614. doi: 10.1091/mbc.e07-06-0581 17804815 PMC2043575

[B7] Chen H. King R. Smith D. Bayon C. Ashfield T. Torriani S.. (2023). Combined pangenomics and transcriptomics reveals core and redundant virulence processes in a rapidly evolving fungal plant pathogen. BMC Biol. 21 (1), 24. doi: 10.1186/s12915-023-01520-6 36747219 PMC9903594

[B8] Fantozzi E. Kilaru S. Gurr S. J. Steinberg G. (2021). Asynchronous development of *Zymoseptoria tritici* infection in wheat. Fungal Genet. Biol. 146, 103504. doi: 10.1016/j.fgb.2020.103504 33326850 PMC7812371

[B9] Fisher M. C. Henk D. A. Briggs C. J. Brownstein J. S. Madoff L. C. McCraw S. L.. (2012). Emerging fungal threats to animal, plant and ecosystem health. Nature 484 (7393), 186–194. doi: 10.1038/nature10947 22498624 PMC3821985

[B10] Francisco C. S. Ma X. Zwyssig M. M. McDonald B. A. Palma-Guerrero J. (2019). Morphological changes in response to environmental stresses in the fungal plant pathogen *Zymoseptoria tritici* . Sci. Rep. 9 (1), 9642. doi: 10.1038/s41598-019-45994-3 31270361 PMC6610121

[B11] Goodwin S. B. M'Barek S. Dhillon B. Wittenberg A. H. J. Crane C. F. Hane J. K.. (2011). Finished genome of the fungal wheat pathogen *Mycosphaerella graminicola* reveals dispensome structure, chromosome plasticity, and stealth pathogenesis. PloS Genet. 7 (6), e1002070. doi: 10.1371/journal.pgen.1002070 21695235 PMC3111534

[B12] Grandaubert J. Bhattacharyya A. Stukenbrock E. H. (2015). RNA-seq-Based gene annotation and comparative genomics of four fungal grass pathogens in the genus zymoseptoria identify novel orphan genes and species-specific invasions of transposable elements. G3 Genes|Genomes|Genetics 5 (7), 1323–1333. doi: 10.1534/g3.115.017731 25917918 PMC4502367

[B13] Habig M. Quade J. Stukenbrock E. H. (2017). Forward genetics approach reveals host genotype-dependent importance of accessory chromosomes in the fungal wheat pathogen *Zymoseptoria tritici* . MBio 8 (6), e01919-17. doi: 10.1128/mBio.01919-17 29184021 PMC5705923

[B14] Han J. Lee J.-D. Bibbs L. Ulevitch R. J. (1994). A MAP kinase targeted by endotoxin and hyperosmolarity in mammalian cells. Science 265 (5173), 808–811. doi: 10.1126/science.7914033 7914033

[B15] Hassani-Pak K. Singh A. Brandizi M. Hearnshaw J. Parsons J. D. Amberkar S.. (2021). KnetMiner: a comprehensive approach for supporting evidence-based gene discovery and complex trait analysis across species. Plant Biotechnol. J. 19 (8), 1670–1678. doi: 10.1111/pbi.13583 33750020 PMC8384599

[B16] Hodgens C. Chang N. Eric Schaller G. Kieber J. J. (2020). Mutagenomics: A rapid, high-throughput method to identify causative mutations from a genetic screen. Plant Physiol. 184 (4), 1658–1673. doi: 10.1104/pp.20.00609 32887734 PMC7723078

[B17] Hohmann S. (2009). Control of high osmolarity signalling in the yeast saccharomyces cerevisiae. FEBS Lett. 583 (24), 4025–4029. doi: 10.1016/j.febslet.2009.10.069 19878680

[B18] Kahmann R. Basse C. (1999). REMI (Restriction enzyme mediated integration) and its impact on the isolation of pathogenicity genes in fungi attacking plants. Eur. J. Plant Pathol. 105 (3), 221–229. doi: 10.1023/A:1008757414036

[B19] Kellner R. Bhattacharyya A. Poppe S. Hsu T. Y. Brem R. B. Stukenbrock E. H. (2014). Expression profiling of the wheat pathogen *Zymoseptoria tritici* reveals genomic patterns of transcription and host-specific regulatory programs. Genome Biol. Evol. 6 (6), 1353–1365. doi: 10.1093/gbe/evu101 24920004 PMC4079195

[B20] Keon J. Antoniw J. Carzaniga R. Deller S. Ward J. L. Baker J. M.. (2007). Transcriptional adaptation of *Mycosphaerella graminicola* to programmed cell death (PCD) of its susceptible wheat host. Mol. Plant-Microbe Interact. 20 (2), 178–193. doi: 10.1094/MPMI-20-2-0178 17313169

[B21] Kilaru S. Fantozzi E. Cannon S. Schuster M. Chaloner T. M. Guiu-Aragones C.. (2022). *Zymoseptoria tritici* white-collar complex integrates light, temperature and plant cues to initiate dimorphism and pathogenesis. Nat. Commun. 13 (1), 5625. doi: 10.1038/s41467-022-33183-2 36163135 PMC9512790

[B22] Kim D. Paggi J. M. Park C. Bennett C. Salzberg S. L. (2019). Graph-based genome alignment and genotyping with HISAT2 and HISAT-genotype. Nat. Biotechnol. 37 (8), 907–915. doi: 10.1038/s41587-019-0201-4 31375807 PMC7605509

[B23] King R. Urban M. Lauder R. P. Hawkins N. Evans M. Plummer A.. (2017). A conserved fungal glycosyltransferase facilitates pathogenesis of plants by enabling hyphal growth on solid surfaces. PloS Pathog. 13 (10), e1006672. doi: 10.1371/journal.ppat.1006672 29020037 PMC5653360

[B24] Kramer B. Thines E. Foster A. J. (2009). MAP kinase signalling pathway components and targets conserved between the distantly related plant pathogenic fungi *Mycosphaerella graminicola* and *Magnaporthe grisea* . Fungal Genet. Biol. 46 (9), 667–681. doi: 10.1016/j.fgb.2009.06.001 19520179

[B25] Lendenmann M. H. Croll D. Stewart E. L. McDonald B. A. (2014). Quantitative trait locus mapping of melanization in the plant pathogenic fungus *Zymoseptoria tritici* . Genes|Genomes|Genetics 4 (12), 2519–2533. doi: 10.1534/g3.114.015289 25360032 PMC4267946

[B26] Lengeler K. B. Davidson R. C. D'souza C. Harashima T. Shen W.-C. Wang P.. (2000). Signal transduction cascades regulating fungal development and virulence. Microbiol. Mol. Biol. Rev. 64 (4), 746–785. doi: 10.1128/MMBR.64.4.746-785.2000 11104818 PMC99013

[B27] Liao Y. Smyth G. K. Shi W. (2014). FeatureCounts: An efficient general purpose program for assigning sequence reads to genomic features. Bioinformatics 30 (7), 923–930. doi: 10.1093/bioinformatics/btt656 24227677

[B28] Love M. I. Huber W. Anders S. (2014). Moderated estimation of fold change and dispersion for RNA-seq data with DESeq2. Genome Biol. 15 (12), 1–21. doi: 10.1186/s13059-014-0550-8 PMC4302049 25516281

[B29] Maeda T. Takekawa M. Saito H. (1995). Activation of yeast PBS2 MAPKK by MAPKKKs or by binding of an SH3-containing osmosensor. Science 269 (5223), 554–558. doi: 10.1126/science.7624781 7624781

[B30] Maier F. J. Schäfer W. (1999). Mutagenesis *via* insertional or restriction enzyme-mediated integration (REMI) as a tool to tag pathogenicity related genes in plant pathogenic fungi. Biol. Chem. 380 (7–8), 855–864. doi: 10.1515/BC.1999.105 10494834

[B31] McCorison C. B. Goodwin S. B. (2020). The wheat pathogen *Zymoseptoria tritici* senses and responds to different wavelengths of light. BMC Genomics 21 (1), 1–15. doi: 10.1186/s12864-020-06899-y PMC7382159 32711450

[B32] McDonald B. A. Martinez J. P. (1991). Chromosome length polymorphisms in a septoria tritici population. Curr. Genet. 19 (4), 265–271. doi: 10.1007/BF00355053

[B33] McDonald M. C. McGinness L. Hane J. K. Williams A. H. Milgate A. Solomon P. S. (2016). Utilising gene tree variation to identify candidate effector genes in *Zymoseptoria tritici* . G3: Genes Genomes Genet. 6 (4), 779–791. doi: 10.1534/g3.115.025197 PMC4825649 26837952

[B34] Mehrabi R. van der Lee T. Waalwijk C. Gert H. J. K. (2006a). MgSlt2, a cellular integrity MAP kinase gene of the fungal wheat pathogen mycosphaerella graminicola, is dispensable for penetration but essential for invasive growth. Mol. Plant-Microbe Interact. 19 (4), 389–398. doi: 10.1094/MPMI-19-0389 16610742

[B35] Mehrabi R. Zwiers L.-H. de Waard M. A. Kema G. H. J. (2006b). MgHog1 regulates dimorphism and pathogenicity in the fungal wheat pathogen *Mycosphaerella graminicola* . Mol. Plant-Microbe Interact. 19 (11), 1262–1269. doi: 10.1094/MPMI-19-1262 17073308

[B36] Meile L. Croll D. Brunner P. C. Plissonneau C. Hartmann F. E. McDonald B. A.. (2018). A fungal avirulence factor encoded in a highly plastic genomic region triggers partial resistance to septoria tritici blotch. New Phytol. 219 (3), 1048–1061. doi: 10.1111/nph.15180 29693722 PMC6055703

[B37] Michielse C. B. Hooykaas P. J. J. van den Hondel C. A. M. J. J. Ram A. F. J. (2005). *Agrobacterium*-mediated transformation as a tool for functional genomics in fungi. Curr. Genet. 48 (1), 1–17. doi: 10.1007/s00294-005-0578-0 15889258

[B38] Mohammadi N. Mehrabi R. Mirzadi Gohari A. Roostaei M. Mohammadi Goltapeh E. Safaie N.. (2020). MADS-box transcription factor ZtRlm1 is responsible for virulence and development of the fungal wheat pathogen *Zymoseptoria tritici* . Front. Microbiol. 11. doi: 10.3389/fmicb.2020.01976 PMC7461931 33013739

[B39] Möller M. Habig M. Freitag M. Stukenbrock E. H. (2018). Extraordinary genome instability and widespread chromosome rearrangements during vegetative growth. Genetics 210 (2), 517–529. doi: 10.1534/genetics.118.301050 30072376 PMC6216587

[B40] Motteram J. Küfner I. Deller S. Brunner F. Hammond-Kosack K. E. Nürnberger T.. (2009). Molecular characterisation and functional analysis of MgNLP, the sole NPP1 domain-containing protein, from the fungal wheat leaf pathogen *Mycosphaerella graminicola* . Mol. Plant-Microbe Interact. 22 (7), 790–799. doi: 10.1094/MPMI-22-7-0790 19522561

[B41] Motteram J. Lovegrove A. Pirie E. Marsh J. Devonshire J. van de Meene A.. (2011). Aberrant protein n-glycosylation impacts upon infection-related growth transitions of the haploid plant-pathogenic fungus *Mycosphaerella graminicola* . Mol. Microbiol. 81 (2), 415–433. doi: 10.1111/j.1365-2958.2011.07701.x 21623954

[B42] Mullins E. D. Kang S. (2001). Transformation: a tool for studying fungal pathogens of plants. Cell. Mol. Life Sci. 58 (14), 2043–2052. doi: 10.1007/PL00000835 11814055 PMC11337336

[B43] Nan G.-L. Walbot V. (2009). Plasmid rescue: Recovery of flanking genomic sequences from transgenic transposon insertion sites. In Methods Mol. Biol. 526 (1), 101–109. doi: 10.1007/978-1-59745-494-0_8 19377999

[B44] Oggenfuss U. Badet T. Wicker T. Hartmann F. E. Singh N. K. Abraham L.. (2021). A population-level invasion by transposable elements triggers genome expansion in a fungal pathogen. ELife 10, 1–25. doi: 10.7554/eLife.69249 PMC8445621 34528512

[B45] Østergaard L. Yanofsky M. F. (2004). Establishing gene function by mutagenesis in *Arabidopsis thaliana* . Plant J. 39 (5), 682–696. doi: 10.1111/j.1365-313X.2004.02149.x 15315632

[B46] Palma-Guerrero J. Torriani S. F. F. Zala M. Carter D. Courbot M. Rudd J. J.. (2016). Comparative transcriptomic analyses of *Zymoseptoria tritici* strains show complex lifestyle transitions and intraspecific variability in transcription profiles. Mol. Plant Pathol. 17 (6), 845–859. doi: 10.1111/mpp.12333 26610174 PMC6638511

[B47] Penna S. Jain S. M. (2017). Mutant resources and mutagenomics in crop plants. Emir. J. Food Agric. 29 (9), 651–657. doi: 10.9755/ejfa.2017.v29.i9.86

[B48] Plissonneau C. Hartmann F. E. Croll D. (2018). Pangenome analyses of the wheat pathogen *Zymoseptoria tritici* reveal the structural basis of a highly plastic eukaryotic genome. BMC Biol. 16 (1), 5. doi: 10.1186/s12915-017-0457-4 29325559 PMC5765654

[B49] Raudvere U. Kolberg L. Kuzmin I. Arak T. Adler P. Peterson H.. (2019). g:Profiler: a web server for functional enrichment analysis and conversions of gene lists, (2019 update). Nucleic Acids Res. 47 (W1), W191–W198. doi: 10.1093/nar/gkz369 31066453 PMC6602461

[B50] Rodriguez-Moreno L. Ebert M. K. Bolton M. D. Thomma B. P. H. J. (2018). Tools of the crook-infection strategies of fungal plant pathogens. Plant J. 93 (4), 664–674. doi: 10.1111/tpj.13810 29277938

[B51] Rossman A. Y. Crous P. W. Hyde K. D. Hawksworth D. L. Aptroot A. Bezerra J. L.. (2015). Recommended names for pleomorphic genera in *Dothideomycetes* . IMA Fungus 6 (2), 507–523. doi: 10.5598/imafungus.2015.06.02.14 26734553 PMC4681266

[B52] Rudd J. J. Kanyuka K. Hassani-Pak K. Derbyshire M. Andongabo A. Devonshire J.. (2015). Transcriptome and metabolite profiling of the infection cycle of *Zymoseptoria tritici* on wheat reveals a biphasic interaction with plant immunity involving differential pathogen chromosomal contributions and a variation on the hemibiotrophic lifestyle definition. Plant Physiol. 167 (3), 1158–1185. doi: 10.1104/pp.114.255927 25596183 PMC4348787

[B53] Rudd J. J. Keon J. Hammond-Kosack K. E. (2008). The wheat mitogen-activated protein kinases TaMPK3 and TaMPK6 are differentially regulated at multiple levels during compatible disease interactions with *Mycosphaerella graminicola* . Plant Physiol. 147 (2), 802–815. doi: 10.1104/pp.108.119511 18441220 PMC2409019

[B54] Singer T. Burke E. (2003). “High-throughput TAIL-PCR as a tool to identify DNA flanking insertions.” in Plant Functional Genomics (Humana Press) 236, 241–272. doi: 10.1385/1-59259-413-1:241 14501069

[B55] Singh Y. Nair A. M. Verma P. K. (2021). Surviving the odds: From perception to survival of fungal phytopathogens under host-generated oxidative burst. Plant Commun. 2 (3), 100142. doi: 10.1016/j.xplc.2021.100142 34027389 PMC8132124

[B56] Steinberg G. (2015). Cell biology of *Zymoseptoria tritici*: Pathogen cell organisation and wheat infection. Fungal Genet. Biol. 79, 17–23. doi: 10.1016/j.fgb.2015.04.002 26092785 PMC4502449

[B57] Testa A. Oliver R. Hane J. (2015). Overview of genomic and bioinformatic resources for *Zymoseptoria tritici* . Fungal Genet. Biol. 79, 13–16. doi: 10.1016/j.fgb.2015.04.011 26092784

[B58] Urban M. King R. Hassani-Pak K. Hammond-Kosack K. E. (2015). Whole-genome analysis of *Fusarium graminearum* insertional mutants identifies virulence associated genes and unmasks untagged chromosomal deletions. BMC Genomics 16 (1), 261. doi: 10.1186/s12864-015-1412-9 25881124 PMC4404607

[B59] Weld R. J. Plummer K. M. Carpenter M. A. Ridgway H. J. (2006). Approaches to functional genomics in filamentous fungi. Cell Res. 16 (1), 31–44. doi: 10.1038/sj.cr.7310006 16467874

[B60] Wittenberg A. H. J. van der Lee T. A. J. M’Barek S. B. Ware S. B. Goodwin S. B. Kilian A.. (2009). Meiosis drives extraordinary genome plasticity in the haploid fungal plant pathogen *Mycosphaerella graminicola* . PloS One 4 (6), e5863. doi: 10.1371/journal.pone.0005863 19516898 PMC2689623

[B61] Yemelin A. Brauchler A. Jacob S. Foster A. J. Laufer J. Heck L.. (2021). Two novel dimorphism-related virulence factors of *Zymoseptoria tritici* identified using *Agrobacterium*-mediated insertional mutagenesis. Int. J. Mol. Sci. 23 (1), 400. doi: 10.3390/ijms23010400 35008825 PMC8745584

[B62] Yemelin A. Brauchler A. Jacob S. Laufer J. Heck L. Foster A. J.. (2017). Identification of factors involved in dimorphism and pathogenicity of *Zymoseptoria tritici* . PloS One 12 (8), e0183065. doi: 10.1371/journal.pone.0183065 28829795 PMC5568738

[B63] Zhan J. Kema G. H. Waalwijk C. McDonald B. (2002). Distribution of mating type alleles in the wheat pathogen *Mycosphaerella graminicola* over spatial scales from lesions to continents. Fungal Genet. Biol. 36 (2), 128–136. doi: 10.1016/S1087-1845(02)00013-0 12081466

[B64] Zhong Z. Marcel T. C. Hartmann F. E. Ma X. Plissonneau C. Zala M.. (2017). A small secreted protein in *Zymoseptoria tritici* is responsible for avirulence on wheat cultivars carrying the Stb6 resistance gene. New Phytol. 214 (2), 619–631. doi: 10.1111/nph.14434 28164301

[B65] Zwiers L. H. De Waard M. A. (2001). Efficient *Agrobacterium tumefaciens*-mediated gene disruption in the phytopathogen *Mycosphaerella graminicola* . Curr. Genet. 39 (5–6), 388–393. doi: 10.1007/s002940100216 11525415

